# Monitoring pharmacodynamic and molecular drug targets in liquid biopsy: Exploratory study in liver cancer with modelling of EGFR Receptor engagement

**DOI:** 10.1002/bcp.70674

**Published:** 2026-07-14

**Authors:** Zubida M. Al‐Majdoub, Armin Sepp, Amin Rostami‐Hodjegan, Brahim Achour

**Affiliations:** ^1^ Centre for Applied Pharmacokinetic Research, School of Health Sciences University of Manchester Manchester UK; ^2^ Certara Predictive Technologies Certara UK Sheffield UK; ^3^ Department of Biomedical and Pharmaceutical Sciences, College of Pharmacy the University of Rhode Island Kingston Rhode Island USA

**Keywords:** cancer therapeutics, liquid biopsy, pharmacodynamics, precision dosing, systems pharmacology

## Abstract

**Aim:**

Liquid biopsy is minimally invasive (compared with tissue biopsy) and has previously been used to generate systems data regarding drug elimination via hepatic enzymes and transporters. This study extends quantitative assessment of systems parameters in liquid biopsy to pharmacodynamic (PD) and disease markers relevant to cancer development and therapeutics.

**Methods:**

Plasma extracellular vesicles (EVs) were extracted from 29 liver cancer donors. Cell‐free RNA from EVs was subjected to RNAseq, allowing differential expression of PD/disease markers to be investigated relative to healthy baseline. Proteomics of matched liver membrane fractions was used to assess correlation of hepatic abundance with expression in plasma EVs. As a case example, quantitative systems pharmacology (QSP) modelling was used to evaluate EGFR occupancy with its ligand EGF and the EGFR‐specific mAb, cetuximab.

**Results:**

Expression of 406 PD markers and drug targets was monitored in plasma‐derived EVs (including >130 molecular drug targets and >240 disease markers). Differential expression analysis revealed 18 dysregulated pathways involved in cell growth, proliferation, invasion, apoptosis, angiogenesis and immune regulation. EV expression of 33 markers with corresponding liver abundance lacked correlation, reflecting ubiquitous/extrahepatic expression of PD markers. QSP modelling of EGFR receptor occupancy with standard‐dose cetuximab in the presence of EGF demonstrated suboptimal receptor blockade by the mAb (~80%) in patients with high EGFR expression.

**Conclusion:**

This study demonstrated the utility of liquid biopsy in monitoring PD targets and informing QSP models of disease. The approach may be used to guide drug dosing adjustment based on disease progression or remission.

**What is already known about this subject**

- Liquid biopsy may be used to characterize patients for hepatic elimination capacity by monitoring the expression of hepatic enzymes and transporters.
- Liquid biopsy data can inform physiologically based pharmacokinetic (PBPK) modelling to facilitate achieving uniform exposure across patients.

**What this study adds**

- The study extends the application of liquid biopsy to monitoring the levels of pharmacodynamic markers and molecular drug targets.
- A proof‐of‐concept application of liquid biopsy data with quantitative systems pharmacology (QSP) modelling in liver cancer.

## 1 INTRODUCTION

Drug dosing in cancer chemotherapy often follows a one‐size‐fits‐all approach, in which patients are administered a standard dose in the first cycle of treatment, typically determined by body weight or body surface area.^1^ This practice tends to lead to ineffective dosing or toxicity in certain patient groups at the start of therapy due to variable drug response, which generally correlates with the patient's individual characteristics.^2^, ^3^ Moreover, dosages in oncology are rarely adjusted to reflect temporal changes in pathology, unlike cases where there is an easily accessible marker of pharmacodynamic (PD) response (e.g., blood pressure in cardiovascular disease or blood sugar levels in diabetes). This led to recent calls by patient advocacy groups and drug regulators to apply more stratified or individualized dosing strategies to improve efficacy, reduce adverse reactions and lower medication wastage.^4^

Implementation of precision dosing within a systems pharmacology framework will invariably require effective patient characterization,^5^ which highlights the potential role of minimally invasive biofluid sampling as a practical alternative to tissue biopsies.^2^, ^6^, ^7^, ^8^, ^9^ The use of disease biomarkers detected in biofluids is established in the area of cancer diagnosis, with diagnostic strategies typically characterizing polymorphisms and copy number variant profiles of patients using circulating DNA.^10^ The use of biofluids in precision dosing should go beyond genotyping to assign a quantitative grade of drug exposure and response to each patient receiving treatment based on expression of key disease and therapeutic pathways.^11^

Within the context of drug pharmacokinetics (PK), we have consistently demonstrated that liquid biopsy enables monitoring of cell‐free RNA (cfRNA) corresponding to approximately 500 enzymes and transporters in plasma samples from patients with liver cancer,^12^ cardiovascular disease^13^ and chronic kidney disease.^14^ Notably, liquid biopsy data were correlated with in vivo activity/phenotype of cytochrome P450 enzymes in hospitalized patients.^13^, ^15^ In addition, a 3‐fold reduction in drug exposure variability was projected using liquid biopsy‐informed physiologically based pharmacokinetic (PBPK) modelling in a proof‐of‐concept study with midazolam, highlighting the potential role of liquid biopsy data in model‐informed precision dosing (MIPD).^14^

In this study, we extend the developed methods to PD and disease biomarkers, and demonstrate the utility of liquid biopsy data in informing quantitative systems pharmacology (QSP) models of drug effects using the example of cetuximab interaction with the epidermal growth factor receptor (EGFR). The study also explores limitations of the approach in relation to availability of well‐characterized human samples and the challenges of establishing correlations between expression of circulating mRNA and EGFR tissue abundance.

## 2 METHODS

### 2.1 Matched human plasma and liver samples

The samples and multi‐omics workflow used in this exploratory study were reported previously.^12^ Briefly, matched plasma and liver tissue samples were supplied by the Manchester Royal Infirmary, Manchester, UK, from 29 liver cancer patients hospitalized for liver resection (13 female; aged 44–85 years; 25 metastatic liver cancer of colorectal origin, two hepatocellular carcinoma and two gallbladder/bile duct cancer). Ethical approval was granted by the Northwest Research Ethics Committee (14/NW/1260 and 19/NW/0644). Histologically normal liver tissue adjacent to tumours was sampled, and blood was collected prior to surgery. Healthy plasma from seven donors (two female; aged 23–57 years) was obtained from BioIVT (West Sussex, UK). Table 1 summarizes donor characteristics. Table S1 lists detailed demographic and clinical information related to the 29 liver cancer patients. Table S2 shows demographics of healthy plasma donors. While this study focused on metastatic liver cancer, the technology is applicable to other cancer types.

**TABLE 1:** Summary of the characteristics of plasma and liver tissue sample sets.

Sample set	*n*	Disease cohort	Sex	Age	Ethnicity	Extracted fraction
Healthy plasma	7	Healthy	M (5), F (2)	23–57 years	White (3), Black (1), Hispanic (3)	Extracellular vesicles
Plasma from liver cancer patients	29	Hepatocellular carcinoma (2), gallbladder/bile duct cancer (2) and colorectal liver metastasis (25)	M (16), F (13)	44–85 years	White (25), − (4)	Extracellular vesicles
Histologically normal liver tissue from liver cancer patients	29	Hepatocellular carcinoma (2), gallbladder/bile duct cancer (2) and colorectal liver metastasis (25)	M (16), F (13)	44–85 years	White (25), − (4)	Total membrane fraction/microsomes

*Note*: In parentheses are the numbers of samples in each category; M, male; F, female; −, data not available.

### 2.2 Cell‐free RNA extraction and sequencing

Extraction of plasma extracellular vesicles (EVs) and cfRNA isolation and sequencing were done as described previously.^9^, ^12^, ^13^, ^16^ Briefly, EVs were extracted from 1–3 mL plasma using polymer‐assisted precipitation (ExoQuick, System Biosciences, Palo Alto, CA), followed by gentle centrifugation (1500 *g*, 60 min, 4°C). Extraction of cfRNA from EVs used MagMax Cell‐Free Total Nucleic Acid Isolation Kit (Thermo Fisher Scientific, Austin, TX). Reverse transcription of cfRNA (3.5 μL) was performed using AmpliSeq™ cDNA Synthesis for Illumina (Cambridge, UK), and cDNA (5 μL) was used in target amplification by PCR (16–17 cycles) using AmpliSeq™ Transcriptome Human Gene Expression Panel and AmpliSeq™ HiFi Mix (Illumina). Libraries were prepared for sequencing using AmpliSeq™ Library PLUS (96 reactions), purified and further amplified (7 cycles). Universal primers complementary to adapter flow cell binding sites were used, and the final libraries were purified using Agencourt AMPure XP with size selection (range: 200–700 bp). Normalized libraries were pooled and diluted, followed by sequencing on NovaSeq™ 6000 (Illumina) with 2 × 150 bp paired‐end reads.

Sequencing data were analysed using RNA Amplicon App 2.0.1 (Illumina), alignment employed the Burrows Wheeler Aligner and differential expression analysis used DESeq2.^17^ Transcripts of PD targets were selected using database searches (see *Functional annotation of PD targets*), and expression was recorded relative to the total number of reads in each sample as reads per million (RPM).

### 2.3 Proteomics of liver samples

Proteomic analysis was done as previously reported.^12^ Liver tissue (42─379 mg) was mechanically homogenized, and membrane fractions were prepared by differential centrifugation (10 000 *g* for 20 min, followed by 100 000 *g* for 75 min). Protein content of membrane fractions was measured with the Bradford assay, and 43─50 μg of individual samples (*n* = 29) and a quality control pool (analysed in duplicate) were prepared for proteomics using filter‐aided sample preparation,^18^ with sequential proteolysis with lysyl endopeptidase (1:50 w/w) and trypsin (1:25 w/w). Peptides were analysed using an UltiMate® 3000 rapid separation liquid chromatography system (Dionex, Surrey, UK) connected to an Orbitrap Elite mass spectrometer (Thermo Fisher Scientific, Waltham, MA). Chromatography was carried out over a 90‐min gradient, and eluted peptides were analysed over 350–1500 mass‐to‐charge range. Data‐dependent acquisition was used to automatically select the top 10 multiply charged ions for fragmentation by collision‐induced dissociation.

Proteomic data were processed using MaxQuant 1.6.7.0 (Max Planck Institute, Martinsried, Germany) against a human UniprotKB proteome database (74 788 sequences), with 1% peptide/protein false discovery rates. Protein identification required at least one razor peptide, as described previously,^19^ and detection in at least three (out of 29) samples. Quantification used the total protein approach.^20^, ^21^

### 2.4 Statistical analysis

Microsoft Excel 2016, GraphPad Prism 10.0.2 (La Jolla, CA) and MATLAB 2023b (MathWorks, Natick, MA) were used for statistics and graphical illustration. RNA sequencing data were included in quantitative analysis only if a sample had (i) sufficiently high cfRNA yield (>1 ng/mL plasma) and quality (DV200 score >20%), and (ii) a sufficient sequencing depth (>20 million reads). Data were presented as mean and SD and variability as CV. Analysis included 2–3 replicates per healthy sample (22 replicates in total) and 1 replicate per cancer sample (29 samples); data were used only when at least three measurements were available for a gene in the healthy or disease set. Differences in RNA expression were assessed by an unpaired *t*‐test, assuming unequal variance between groups, with strict criteria (>5‐fold change in mean expression, *p* < 0.01) to account for the small healthy sample size (*n* = 7). Assessment of correlation in expression (Pearson's correlation, *r*) focused on PD targets detected in matched plasma and liver samples with ≥6 sample pairs, with *p*‐value cutoff set at 0.05.

### 2.5 Functional annotation of PD targets

PD targets related to cancer therapeutics and organ health were identified using several databases: Human Protein Atlas (https://www.proteinatlas.org/), UniprotKB (https://www.uniprot.org/), NCBI Gene (https://www.ncbi.nlm.nih.gov/gene), Online Mendelian Inheritance in Man (https://www.omim.org/) and a literature search. The targets were classed into tyrosine and other protein kinases, growth factors and receptors, immune regulation and response factors, apoptotic markers, organ health and homeostasis markers, inflammatory regulators and angiogenesis markers. The Human Protein Atlas (https://www.proteinatlas.org/)^22^ (version 24.0, updated 10/22/2024) was used to assess tissue‐specific enrichment of identified genes, EGFR tissue expression and enriched genes across tissues. UniprotKB was used to assess hepatic cellular localization of target proteins. Ingenuity Pathway Analysis (QIAGEN, https://www.qiagenbioinformatics.com/products/ingenuitypathway-analysis) was used to annotate druggable pathways.

### 2.6 QSP modelling of EGFR engagement

As a case example, a QSP model of EGF and EGFR interaction in the absence or presence of cetuximab was built using the SimBiology toolbox in MATLAB 2023b. An outline of the model is shown in Figure S1. The model was based on the standard full PBPK tissue layout scheme introduced for mAbs by Shah and Betts^23^ and was modified to accommodate two‐pore circulation of proteins of any size, as reported previously by Sepp et al.,^24^ with several modifications, as detailed in Supporting Information. EGF and EGFR mRNA levels in plasma EVs from the healthy and patient subgroups were fitted to lognormal distribution to calculate geometric means and confidence intervals (Figure S2), allowing variability within each cohort and changes in the disease cohort from healthy baseline to be defined. Higher levels of EGF and EGFR mRNA were detected in plasma from cancer patients, whereas weak correlation was observed between individual EGF and EGFR RNA expression data (Figure S3); therefore, EGFR and EGF were not correlated in the model.

The QSP model was used to estimate EGFR receptor occupancy (RO) with EGF or cetuximab under varying EGF and EGFR expression levels, with the mAb at different binding affinities and concentrations (see Supporting Information). It was assumed that variation in EGF and EGFR plasma concentrations is solely due to variation in expression rates, with the receptor shedding rate being postulated to be invariant. Steady‐state EGFR occupancy with EGF is shown in Figure S4 in the absence of the mAb; at normal EGF and EGFR expression levels, EGFR occupancy with its cognate ligand was predicted to be 0.055, but this can reach 0.9 at the highest simulated level of EGF. Given that membrane‐bound EGFR concentrations in organs exceed the Kd value for EGF, this interaction can be described as receptor‐controlled. Five different EGF and EGFR expression levels that cover the observed expression variation around the population mean at four different antibody affinities (nanomolar to picomolar) and four doses (0.5 to 10 mg/kg) were simulated in all possible combinations for steady‐state EGFR occupancy with EGF, the mAb and both EGF and the mAb, as presented in Figures S5, S6 and S7, respectively. Finally, the model was used to simulate a standard dosing regimen based on the package insert of cetuximab (ERBITUX),^25^ as indicated for colorectal cancer (400 mg/m^2^ followed by 250 mg/m^2^ weekly until steady state). Drug exposure and target engagement were simulated (target engagement of >95% is used here as a benchmark, which is considered achievable with the recommended clinical dose of cetuximab). Observed loading dose (400 mg/m^2^) cetuximab concentrations were derived from Fracasso et al.^26^

## 3 RESULTS

### 3.1 Overview of liquid biopsy sequencing output

The concentration of cfRNA in cancer plasma samples was 1.2 ± 0.8 ng/μL (0.4–3 ng/μL), translating to a yield of 7.3 ± 4.4 ng/mL plasma, compared with a yield of 2.2 ± 0.9 ng/mL from healthy plasma. DV200 score was 47% ± 24% for the patient set (and 63% ± 9% in healthy controls). AmpliSeq enabled multiplexed targeted sequencing of >20 000 human RefSeq^27^ genes, generating a read count assigned to each gene in each sample.^12^ Overall, 47–137 million reads were monitored in plasma from patients, compared with 25–96 million reads in healthy controls. Sequencing quality was excellent; 97% of sequenced bases in patient samples (and ≥94% in healthy samples) achieved Q‐scores of ≥30 (i.e., ≥99.9% base call accuracy). The number of gene transcripts measured in patient samples ranged from 18 128 to 21 334, compared with 15 813–19 816 in the healthy set, most of which were protein‐coding genes.

### 3.2 Monitoring of PD and disease markers in liquid biopsy

A total of 171 and 362 gene transcripts of xenobiotic‐metabolizing enzymes and transporters, respectively, were quantifiable in plasma EVs,^12^ in addition to the neonatal Fc receptor, FcRn, implicated in exposure to biologics.^16^ Of these, 89 enzymes and 71 transporters were liver enriched (52.0% and 19.6% of all enzymes and transporters, respectively), as reported previously.^12^ In addition, 406 PD/disease markers were monitored in plasma EVs (Figure 1A, Table 2), including molecular drug targets (*n* = 138), disease markers (*n* = 248) and immune response markers (*n* = 115). A detailed list of PD targets is provided in Table S3. Of the 406 PD targets, only 11 were liver‐enriched (ANGPTL3, ANGPTL6, APOA1, APOA2, APOB, APOC1, APOM, FGF21, IL27, PDIA5 and PNPLA3), which represent 2.7% of monitored targets, indicating ubiquitous expression of PD markers, unlike PK markers for small molecule drugs, which tend to be enriched in the liver, intestine or kidneys. Therefore, normalization with total organism EV cfRNA content was applied instead of organ‐specific cfRNA shedding.^12^, ^15^

**FIGURE 1:**
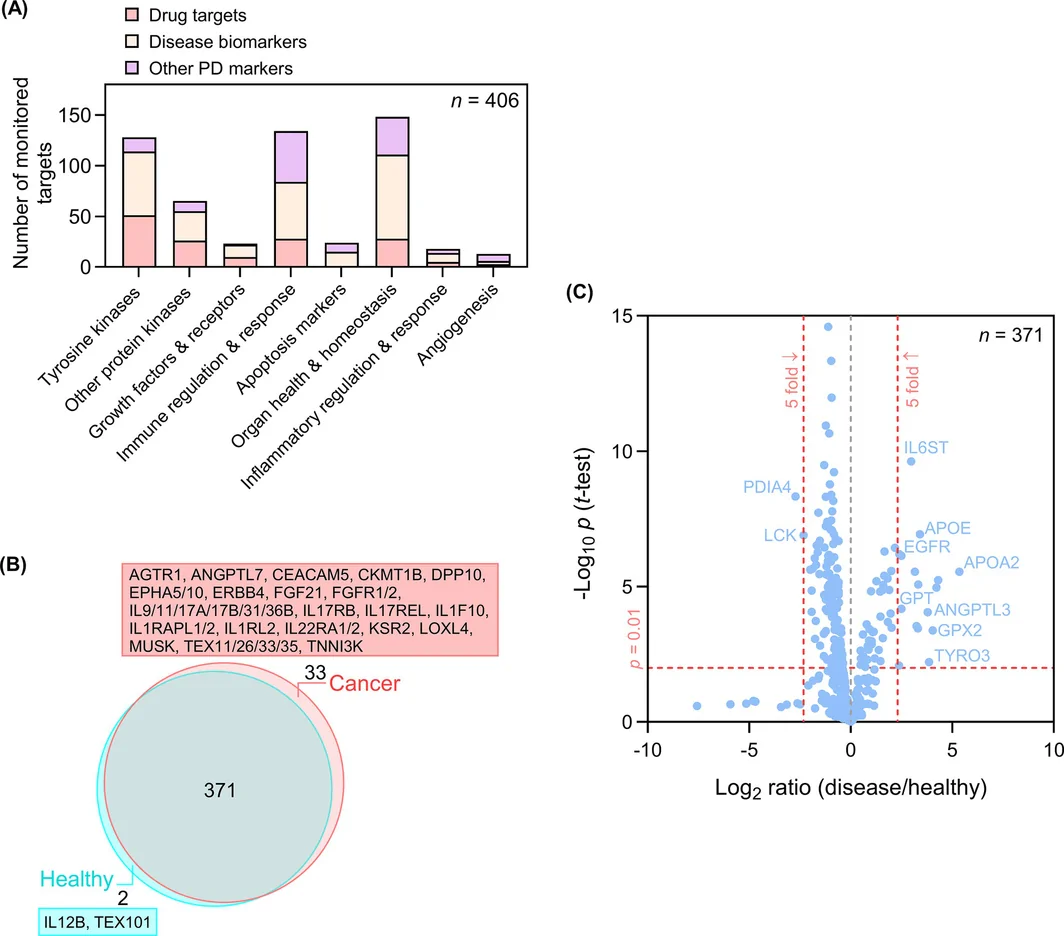
PD and disease markers (*n* = 406) relevant to cancer development and therapeutics monitored in plasma‐derived EVs. Functional analysis defined the functions and molecular class of the markers (list in Table 2, categories overlap) (A). Out of the 406 PD targets, 33 were detected in plasma from cancer patients and two in healthy plasma, whereas 371 were detected in both sets of samples (B). A volcano plot analysis (C) revealed differential expression of PD and disease targets monitored in plasma from cancer patients relative to plasma from healthy donors (*n* = 371 targets); labels are assigned to examples of differentially expressed targets (>5‐fold higher or lower expression in disease, *p* < 0.01 using a *t*‐test with unequal variance, summary in Table 3). Abbreviations*:* EVs, extracellular vesicles; *P*, probability; PD, pharmacodynamic; ↑, upregulated; ↓, downregulated.

**TABLE 2:** List of drug targets, disease markers and pharmacodynamic markers measured in plasma‐derived extracellular vesicles.

Molecular class/function	Drug targets^*a*^	Disease markers^*b*^	Additional PD markers
Tyrosine kinases (cell cycle, proliferation, differentiation, migration and death)	ABL1/2, AXL, BLK, BTK, CSF1R, CSK, DDR1/2, EGFR, EPHA2, ERBB2/3/4, FER, FES, FGR, FLT1/3/4, FRK, FYN, HCK, IGF1R, INSR, JAK1/2/3, KDR, KIT, LCK, LTK, LYN, MERTK, MET, MST1R, NTRK1/3, PDGFRA/B, PTK2/2B/6, RET, SRC, SYK, TEK, TNK2, TYK2, TYRO3 and YES1	ABL1/2, AXL, BLK, BMX, BTK, CSF1R, DDR2, EGFR, EPHA1/2/3/4/7, EPHB2/3/4/6, ERBB2/3/4, FER, FES, FGR, FLT1/3/4, FRK, FYN, HCK, IGF1R, INSR, JAK1/2/3, KDR, KIT, LCK, LTK, LYN, MATK, MERTK, MET, MUSK, NTRK1/3, PDGFRA/B, PTK2/2B/6, RET, SRC, STYK1, SYK, TEK, TIE1, TNK1/2, TYK2, TYRO3, YES1 and ZAP70	AATK, EPHA5/10, EPHB1, INSRR, LMTK2/3, PKDCC, PTK7, ROR1/2, RYK, TEC and TXK
Other protein kinases (cell cycle, proliferation, differentiation, migration and death)	AKT1/2/3, ARAF, BRAF, CDK4/6, MAP2K1/2, MAPK1/3/8/9/10/11, MTOR, PIK3CA, PRKCA/B/D/E/H/I/Q/Z and RAF1	ARAF, AURKA, BUB1, BUB1B, ILK, MAPK1/3/7/8/10/14, PAK1/3, PLK1/2/4, PRKCA/B/D/E/H/I/Q/Z, KSR1/2, TGFBR1, TNNI3K and ZAK	MAP3K5, MAPK4/6/12/13/15, PAK2/4/6 and PLK3
Growth factors and receptors	ABL1, EGFR, ERBB2/4, FGFR1–4 and PDGFRA/B	ABL1/2, EGFR, ERBB2/3/4, FGF21, FGFR1–4 and IGF2R	RYK
Immune regulation and response	CXCR4, DPP4, IL1B/5/6/12B/17A/23A, IL1R1, IL17RA, IL2RA/B/G, IL3RA, IL4R, IL5RA, IL6R, IL11RA, IL23R, ITK, NR1H4, PDCD1 and TLR2/3/4/7/8/9	CXCR1/2/4/5/6/7, DPP4/7/9, IFITM4P, IL1B/2/4/5/6/6ST/7/8/9/10/11/12A/12B/15/16/17A/17B/18/21, IL1R1/2, IL1RAPL1, IL1RN, IL2RA/B/G, IL4R, IL6R, IL7R, IL10RA, IL11RA, IL12RB1, IL13RA1/2, IL17RD, IL21R, IL31RA, ILF3, ITK, LGALS3, NR1H4, PDCD1, PLCG1 and TLR2/4/5	B2M, FCGRT, IFITM1/2/3/10, IL1F10, IL3/4I1/17C/19/25/26/27/31/32/33/34/36A/36B/37, IL1RAPL2, IL1RL1/2, IL9R, IL10RB, IL12RB2, IL15RA, IL17RB/C/E/EL, IL18R1, IL18RAP, IL20RA/B, IL21R‐AS1, IL22RA1/2, IL27RA, IL28RA, ILF2, IRAK4, LGALS1/2/8/9 and TLR1/6/10
Apoptosis markers	PDCD1	BAX, CASP1–10, LGALS4, PDCD1 and TP53	DPP8, LGALS1/7B/9B/9C/12/14/17A and LGALSL
Organ health and homeostasis	AGTR1, APOB, CEACAM5, CKB, CKMT1A/1B/2, CCND1, DPP4, GLP1R, GSR, HMGCR, INSR, K‐RAS, MC4R, NPC1L1, NPR1/2/3, NR3C2, PARP1/2, PGR, PLA2G2A, PPARA/D/G, STAT3 and THRB	AGTR1, ALPL, ALPI, APC, APOA1/A2/B/C1/E/M, ARID1A, AXIN1/2, BRCA1/2, CAV1/2, CCNE1, CDKN2A, CEACAM5, KRT19, CKB, CKMT1A/1B/2, CCND1, CTNNB1, DLC1, DPP3/4/9/10, EP300, FLCN, GLP1R, GOT1/2, GPT, GPT2, GPX2/4, GSR, HMGCR, INSR, HRAS, NRAS, KSR2, LOXL1/3, MC4R, MCC, MLH3, MSH2, MUC1, MYC, NPR2, NR3C2, P4HB, PDGFRL, PGR, PLA2G2A, PLCG1, PMS1/2, PNPLA3/6, PPARG, PTEN, PTPN12, RAD54B, SERPINH1, SEPT9, STAT1/2/3/4/5A/5B/6, STRAP, TEX11/15, THRB and TNC	ACACA/B, AGTRAP, CAVIN1/2, DPP8, GPBAR1, GPX1/3/7/8, GSG2, INSRR, LOXL2/4, NR5A2, PDIA3/4/5/6, PTPRJ, SCD, TEX2/9/10/14/22/26/28/29/30/33/35/101/261/264 and TMX3
Inflammatory regulation and response	NR1H4 and TLR2/4/7/9	CASP1/4/5, LOXL3, NR1H4 and TLR2/3/4/5	TLR1/6/8/10
Angiogenesis	VEGFB, ANGPTL3 and AOC3	TNC and VEGFB/C	ANGPTL1/2/4/5/6/7 and AOC2

*Note*: Identification was based on database searches (Human Protein Atlas, UniProtKB, NCBI Gene, Online Mendelian Inheritance in Man) and additional literature search.

^*a*^ listed markers are US FDA‐approved drug targets.

^*b*^ listed markers have a confirmed link to diagnosis, prognosis or monitoring of disease.

### 3.3 Differential expression of PD and disease markers in liver cancer

Out of 406 PD targets, 33 were exclusively identified in plasma from the cancer patients cohort, while two were exclusively identified in healthy plasma (Figure 1B). The remaining 371 PD targets were identified in both sets, allowing differential expression to be investigated (Table S3); 18 targets had significantly altered expression (fold change >5 or <0.2; *p* < 0.01) (Figure 1C). The function of differentially expressed (16 upregulated and two downregulated) targets was assessed for relevance to disease development, progression and drug therapy. The dysregulated targets are involved in cell growth, survival, migration, apoptosis, immune regulation, organ health and angiogenesis; eight of the differentially expressed markers are established drug targets (Table 3).

**TABLE 3:** Changes in gene expression in cancer from healthy baseline measured in liquid biopsy (fold change in disease relative to healthy baseline). Upregulated targets were mapped to functions that promote cancer development (metastasis, survival migration, angiogenesis, tissue damage, desmoplasia, cell growth and suppression of immune response). Downregulated targets are involved in immune response and organ health/homeostasis.

Upregulated genes			
Gene(s)	Fold change	*p*‐value	Function
GPX2	16.5	0.0004	Redox protection
TYRO3^a^	14.6	0.006	Receptor tyrosine kinase
ANGPTL3^a^	13.9	8.8 × 10^−5^	Angiogenesis
GPT	10	8.4 × 10^−6^	Marker of liver damage
NR1H4^a^	9.9	0.0003	Immune response
PDGFRA^a^	9.5	0.0003	Cell proliferation
IL6ST	7.9	2.4 × 10^−10^	Oncogene
CAVIN2	5.7	7.4 × 10^−7^	Promotes desmoplasia
MAPK10^a^	5.7	6.7 × 10^−5^	Proliferation/differentiation/migration
EGFR^a^	5.3	6.4 × 10^−7^	Growth factor, receptor tyrosine kinase
IL1R2	5.2	0.008	Cytokine receptor; inhibits activity of interleukins (immunosuppression)
APOA1, APOA2, APOB^a^, APOC1, and APOE	>9.0	<0.001	Lipid homeostasis and transport; used in early cancer detection

Downregulated genes			
Gene(s)	Fold change	*p*‐value	Function
LCK^a^	0.20	1.3 × 10^−7^	Pro‐T‐cell immune response
PDIA4	0.15	4.7 × 10^−9^	Involved in protein folding

*Note*: The table lists targets that showed a fold change of at least fivefold with *p* < 0.01 were considered (refer to Figure 1C). Targets detected in cancer samples only (AGTR1, ANGPTL7, CEACAM5, CKMT1B, DPP10, EPHA5/10, ERBB4, FGF21, FGFR1/2, IL9/11/17A/17B/31/36B, IL17RB, IL17REL, IL1F10, IL1RAPL1/2, IL1RL2, IL22RA1/2, KSR2, LOXL4, MUSK, TEX11/26/33/35 and TNNI3K) or in healthy samples only (IL12B and TEX101) are not listed.

^a^ Molecular targets of existing FDA‐approved drugs. Drug targets are identified using Qiagen's Ingenuity Pathway Analysis tool. ANGPTL3, APOA1, APOA2, APOB and APOC1 are liver enriched, and trends may change after correction for liver shedding of EVs into the bloodstream.

### 3.4 Quantitative proteomics of PD markers in liver tissue

Global proteomics identified a total of 18 829 peptides, translating to quantification of 2090 proteins/protein groups (average 1280 ± 210 per sample), with eight peptides on average assigned to each protein and 23.2% average protein sequence coverage. Excellent agreement was recorded between abundance values in replicate quality control samples (Figure S8). Quantified proteins included 88 enzymes and 59 transporters relevant to drug PK, as reported previously,^12^ in addition to FcRn (gene: FCGRT).^16^ Furthermore, a total of 44 cancer‐related PD/disease marker proteins were identified in liver membrane samples (compared with 404 PD markers identified as RNA transcripts in plasma EVs), 36 of which had membrane compartment localization (Table S4). The only quantified liver‐enriched proteins were four secreted apolipoproteins, APOA1/A2/B/C1 (9.1% of targets quantified at the protein level); further confirming the ubiquitous expression of PD targets.

Assessment of correlation between liquid biopsy and liver expression focused on 33 PD targets detected in both plasma and liver membrane fractions, with membrane localization, that returned at least six sample pairs with reliable quantification. Correlations were tested using protein abundance in liver membrane fractions^12^; correlations were moderate for IGF2R, GPX1, GSR, MAP2K1/2 and STAT3 (Pearson's *r* ≥ 0.4) and generally weak for apolipoprotein E, EGFR, ILF3 and LGALS4 (Pearson's *r* < 0.4). No correlation was observed for the rest of the PD targets quantified in matched liver and plasma samples. The observed correlation trends may be partially due to the ubiquitous, extrahepatic expression of the targets, and partly because several targets are not membrane localized, such as apolipoproteins (Table S4).

### 3.5 EGFR and EGF monitoring in plasma‐derived extracellular vesicles

As a case example, EGFR and its prototypic ligand EGF were monitored in plasma‐derived EVs from healthy donors (*n* = 7) and cancer patients (*n* = 29) (Figure 2, Figure S2). Expression of EGFR and EGF followed lognormal distribution in both cohorts (Figure S2). EGFR and EGF levels were 5‐fold higher (unpaired *t*‐test with unequal variance, *p* < 0.001) in cancer samples (Figure S2, Figure 2C), and they correlated weakly (Pearson's *r* = 0.27, *p* = 0.08; Figure S3). EGFR is expressed ubiquitously across more than 50 tissues in the human body (Human Protein Atlas consensus data, Figure 2A), with different levels of shedding into the bloodstream (in‐house data, Figure 2B), and consequently, EGFR levels in EVs had a weak correlation with EGFR protein abundance in matching liver samples from patients with metastatic colorectal carcinoma (*r* = 0.24, Figure 2D). Nonetheless, higher relative EGFR abundance in liver membrane seemed to be associated with higher EGFR expression in plasma EVs (Figure 2E).

**FIGURE 2:**
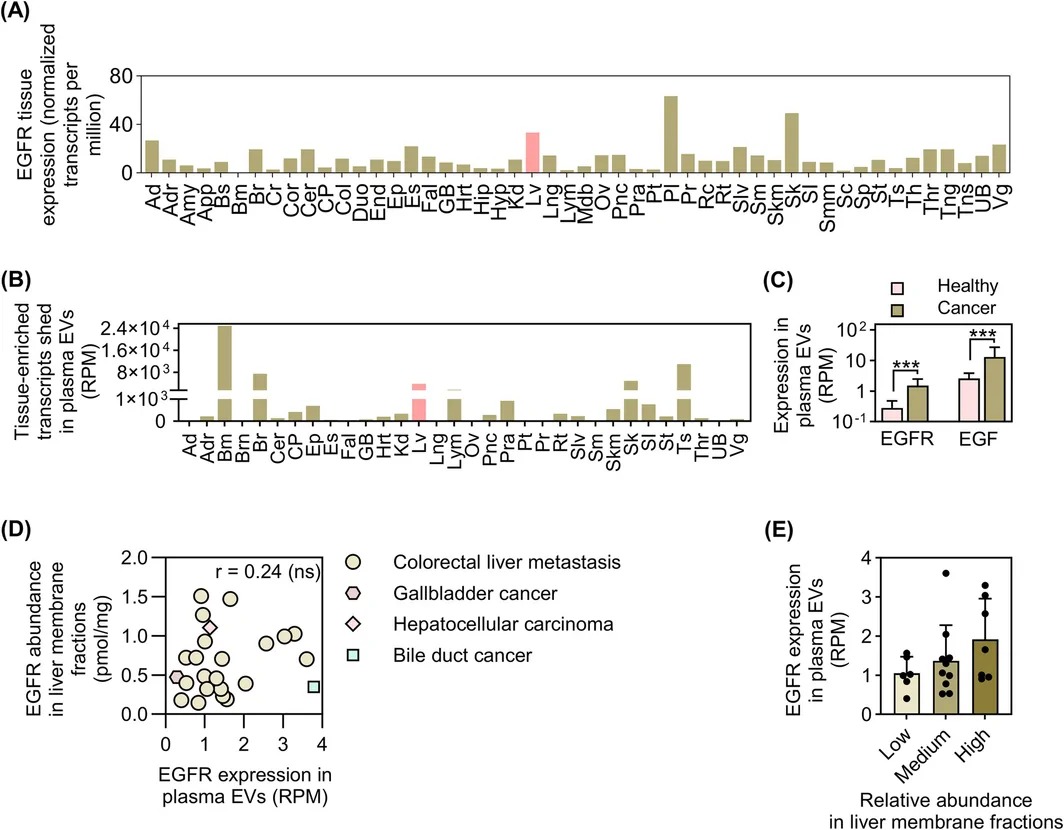
RNA expression of EGFR across 50 different tissues based on consensus data from the Human Protein Atlas (A), including liver, kidneys, skin and intestine, showing ubiquitous expression of EGFR. Average normalized expression of tissue‐enriched transcripts shed in plasma EVs in the cancer cohort using in‐house data (B). Differential expression of EGF and EGFR in cancer and healthy cohorts (C). Correlation between EGFR expression in EVs from plasma and protein abundance in matching liver membrane fractions (D), with samples sorted by diagnosis. Relationship between relative abundance of EGFR in liver membrane fractions (lower quartile to higher quartile) from patients with colorectal liver metastases and expression in matching plasma‐derived EVs (E). In Panel C, ***, *p* < 0.001 using an unpaired *t*‐test with unequal variance. In Panel D, *r* is Pearson's correlation coefficient; ns, nonsignificant (for samples with colorectal liver metastases). Abbreviations: Ad, adipose; Adr, adrenal gland; Amy, amygdala, App, appendix; Bs, basal ganglia; Bm, bone marrow; Br, breast; Brn, brain; Cr, cerebellum, Cor, cerebral cortex; Cer, cervix; CP, choroid plexus; Col, colon; Duo, duodenum; End, endometrium; Ep, epididymis; Es, oesophagus; EVs, extracellular vesicles; Fal, fallopian tube; GB, gallbladder; Hip, hippocampal formation; Hrt; heart muscle; Hyp, hypothalamus; Kd, kidneys; Lng, lungs; Lv, liver; Lym, lymph node/tissue; Mdb, midbrain; Ov, ovary; Pnc, pancreas; Pl, placenta; Pr, prostate; Pra, parathyroid gland; Pt, pituitary gland; Rc, rectum; RPM, reads per million; Rt, retina; Sc, spinal cord; SI, small intestine; Sk, skin; Skm, skeletal muscle; Slv, salivary glands; Sm, seminal vesicle; Smm, smooth muscle; Sp, spleen; St, stomach; Th, thymus; Thr, thyroid glands; Tng, tongue; Tns, tonsil; Ts, testis; UB, urinary bladder; Vg, vagina.

### 3.6 QSP modelling of EGFR receptor occupancy with EGF and cetuximab

A QSP model parameterized with literature data and liquid biopsy quantitative output (although variability between samples was considerable) was used to predict EGFR RO with its ligand EGF and/or cetuximab at different levels of EGF and EGFR, which cover the changes in expression in cancer from baseline, as quantified in the present study. Simulated EGFR RO with cetuximab in the presence of EGF was >99% in the vascular compartment (Figure 3), liver tissue and tumour site (Figure 4), in the tested cases of average baseline healthy and average cancer expression of EGF and EGFR. RO of EGFR with the mAb dropped to approximately 80% on average in extreme cases of high EGFR expression in cancer, indicating a 2‐fold dose increase may be required in these simulated scenarios for higher expressing patients, toxicity permitting (Figure 4). Simulations suggest that assessing soluble EGFR expression in cancer patients may be beneficial prior to cetuximab dosing.

**FIGURE 3:**
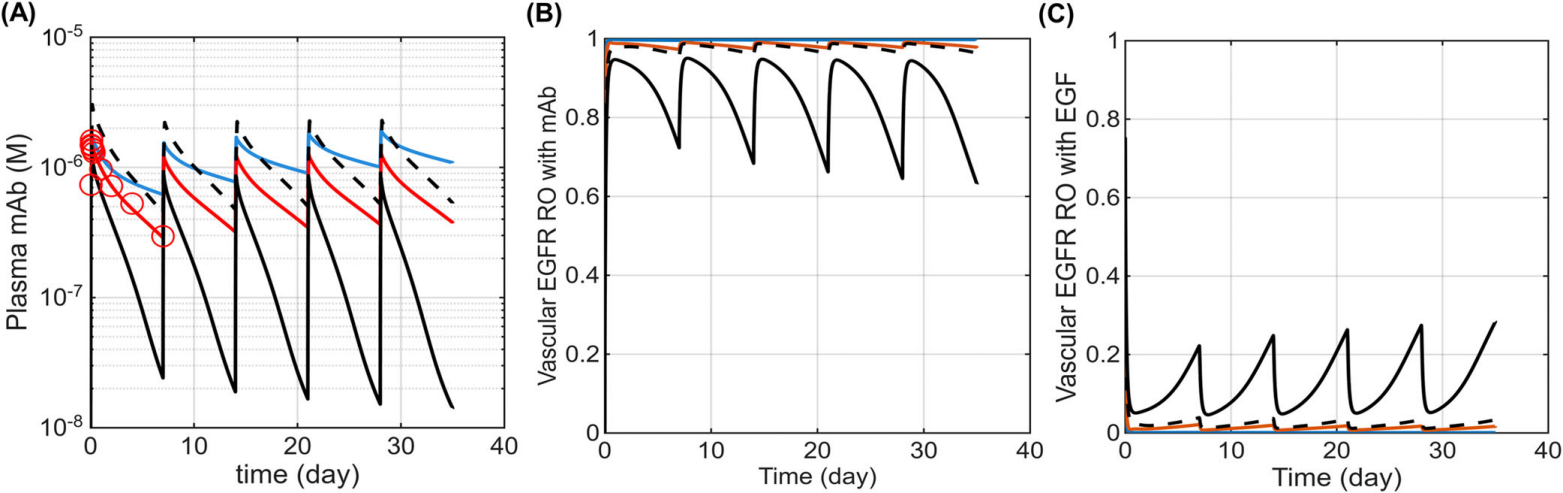
Plasma molar concentration of cetuximab (A) and its effect on vascular endothelial EGFR receptor occupancy (RO) with the mAb (B) or the natural ligand EGF in the presence of the mAb (C), following standard therapeutic dosing for colorectal cancer (400 mg/m^2^ followed by 250 mg/m^2^ weekly). RO is presented as a ratio. Simulations were carried out in different scenarios based on expression data of EGFR and EGF: average healthy expression (blue lines), average expression in cancer (red lines) and high expression in cancer (continuous and dashed black lines). Simulations were done following standard therapeutic dosing for colorectal cancer (400 mg/m^2^, followed by 250 mg/m^2^ weekly) in cases of average healthy (blue lines) and average cancer EGFR expression (red lines). In the case of high cancer EGFR expression, two maintenance dosing regimens were simulated, 250 mg/m^2^ weekly (continuous black lines) and 500 mg/m^2^ weekly (dashed black lines). In Panel A, observed values of mAb plasma concentration for average cancer EGFR expression after a dose of 400 mg/m^2^ (red symbols) were derived from a single‐dose cetuximab clinical study (Fracasso et al.^26^). Abbreviations: mAb, monoclonal antibody.

**FIGURE 4:**
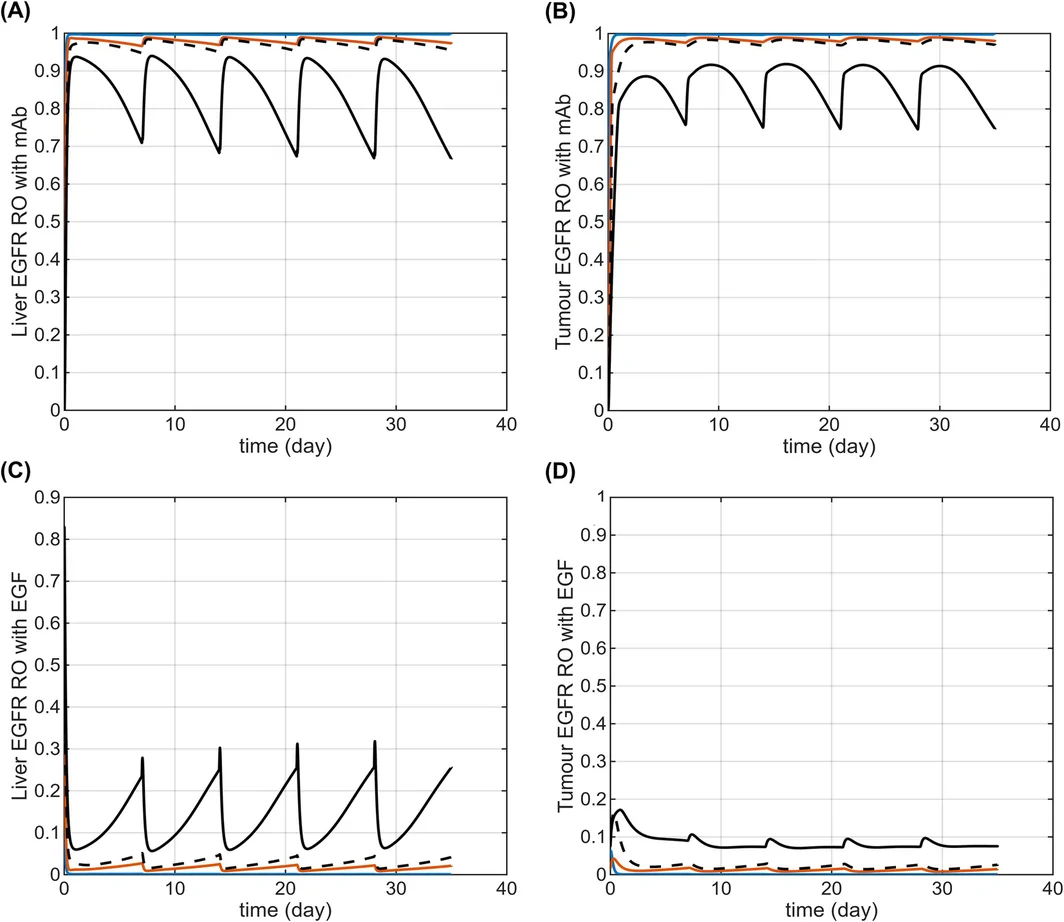
EGFR receptor occupancy (RO) with the mAb cetuximab (A,B) or the biological ligand EGF in the presence of the mAb (C,D), in the liver (A,C) and in the tumour interstitium (B,D), following standard therapeutic dosing for colorectal cancer (400 mg/m^2^ followed by 250 mg/m^2^ weekly). RO is presented as a ratio. Simulations were carried out in different scenarios based on expression data of EGFR and EGF: average healthy expression (blue lines), average expression in cancer (red lines) and high expression in cancer (continuous and dashed black lines). Simulations were done following standard therapeutic dosing for colorectal cancer (400 mg/m^2^, followed by 250 mg/m^2^ weekly) in cases of average healthy (blue lines) and average cancer EGFR expression (red lines). In the case of high cancer EGFR expression, two maintenance dosing regimens were simulated, 250 mg/m^2^ (continuous black lines) and 500 mg/m^2^ (dashed black lines). Abbreviations: mAb, monoclonal antibody.

## 4 DISCUSSION

MIPD has historically been applied with drug‐specific therapeutic drug monitoring (TDM), followed by loops of Bayesian adjustment toward a target drug concentration. Such approach is drug‐specific, and as the number of drugs used in clinical settings increases, the requirement for continuous analytical capability to support TDM becomes challenging if not impossible. Although centralization of TDM application for certain drugs offers a partial remedy (e.g., for clozapine or tacrolimus), this does not address the scale of the problem.^2^ Shifting the focus from the drug to the patient provides a new alternative, or a complementary approach, to TDM, which is not specific to the drug and provides sets of systems data usable for dose individualization. The model‐projected fate of a drug in an individual is assessed against the requirement for a certain drug level, taking into account the characteristics of the disease and the drug targets (addressing between‐individual variations as well as temporal changes in an individual patient over the course of disease/therapy).^5^

Liquid biopsy tests used in cancer diagnostics are not new, and they typically assess differences in polymorphic and copy number variants in cancer patients from baseline using cell‐based or cell‐free DNA extracted from blood samples.^10^ By contrast, assaying cell‐free RNA isolated from plasma‐derived EVs allows for a quantitative grade to be assigned to each patient in relation to pharmacologically relevant pathways, as demonstrated previously in our work^12^, ^13^, ^14^, ^15^ and that of others.^8^, ^28^ Using previously established liquid biopsy assays,^12^ we extend the analysis herein to PD markers relevant to cancer development and therapeutics. These measurements are intended to inform QSP models in combination with complementary data, such as pharmacogenomics and activity phenotypes, in order to gain insight into the aetiology of the disease and response to therapeutic intervention.^2^ The advantage of implementing liquid biopsy‐RNAseq data, as argued previously,^11^ is highlighted in its broad coverage of both PK and PD pathways, allowing quantitative monitoring of a large number of PK (*n* = 533)^12^ and PD (*n* = 404) targets in the liver cancer cohort. Mechanistic models may incorporate the collected data and associated variability when considering the propagation of drug exposure variability into changes in drug effects. Notably, the findings suggest that liquid biopsy screening can extend to the assessment of FDA‐approved drug targets, as reported previously in the contexts of cardiovascular disease^13^ and chronic kidney disease.^14^ Correlation against liver tissue abundance revealed weak‐to‐moderate relationships, including that of EGFR, indicating that the liver was not the main source of the shed EV‐derived PD markers. Ubiquitous expression of PD and drug targets is a key consideration when assessing the source tissue of clinical biomarkers. The primary site of the malignancy (the colon in this case) should also be considered as a contributor to the overall secreted PD target, EGFR, into plasma EVs. The level of EGFR that is important in the case of colorectal liver metastases is that expressed at the tumour site, which is the target of tyrosine kinase inhibitors and mAb‐based therapy. Ideally, it would be beneficial to collect additional data on cancer progression against EGF and EGFR expression levels in relevant tissues and plasma‐derived EVs by proteomics and transcriptomics. If significant correlations were to be observed, optimized antibody dosing could be considered.

The delivery of precise dosing at the point of care highlights one of the most promising clinical applications of liquid biopsy technology.^2^ The utility of individualization with this approach, when applied with PBPK modelling, has been demonstrated in the projected reduction in drug exposure variability reported previously for a CYP3A substrate, midazolam.^14^ It is important to note, however, that variability in exposure is only one factor contributing to variability in response, typically also reflected in variability in receptor occupancy and downstream effects. PD variability is often as, if not more, important than PK variability, but it is less well‐understood in the context of comorbidities and disease heterogeneity.^11^, ^29^ To demonstrate the feasibility of dose stratification using receptor occupancy modelling, we developed a liquid biopsy‐informed QSP model to simulate pharmacological blockade of the EGFR pathway, central to the development of a range of solid tumours, including colorectal cancer and its liver metastases. The simulated drug, cetuximab, is indicated for the treatment of K‐RAS wild‐type, EGFR‐expressing, metastatic colorectal cancer.^25^ Receptor occupancy of EGFR with cetuximab, simulated in the presence of the ligand EGF, dropped from >99% (required for therapeutic efficacy) in an average cancer patient with average expression of EGF and EGFR, to ~80% at higher EGFR expression, indicating subtherapeutic drug‐receptor interaction in high expressors. Modelling downstream signalling pathways and their variations could provide further insights in relation to PD effects, but it was beyond the scope of this work. Importantly, the findings support assessing EGFR expression prior to initiating treatment, in line with existing FDA guidance for cetuximab.^25^ This, however, is largely not implemented in practice or recommended by professional oncology society guidelines for metastatic colorectal cancer, while testing for K‐RAS mutations remains the main factor considered in clinical decision making.^30^

Despite the apparent simplicity of the central dogma of molecular biology, protein‐mRNA abundance correlation remains challenging.^31^ This is generally variable, with considerable variation depending on the gene, tissue, and experimental conditions. In the case of liquid biopsy, the requisite for correction for shedding variability requires the target to be enriched in a certain organ, such as the liver,^6^, ^15^ which is not the case for PD markers, generally characterized by ubiquitous expression. This is especially challenging in the context of mechanistic modelling, which relies on mass action formalism of chemical kinetics, with the rates of the reaction depending on the absolute molar concentrations of the reactants. For example, while EGF and EGFR cfRNA was higher in plasma from cancer patients relative to healthy donors, only a weak correlation was observed for the individual data. Although this may indicate a lack of correlation between the levels of the two proteins and independent gene regulation mechanisms, there is also a possibility that a relationship might exist, which may be masked by the mRNA‐protein apparent irregularity and the added variability in EV shedding.

Whereas the significance of the collected data for modelling PD involvement in drug response variability is emphasized, the reliance on an opportunistic study introduces limitations to the generalizability of the findings, particularly in relation to the limited sample size of the study cohort. In addition, modelling was based on disease‐driven changes in expression in plasma‐derived EVs from healthy baseline, while assuming proportional variation of expression in the tumour, in the absence of such data. Modelling comes with the caveat that levels are based on measured protein concentrations, and inferences from mRNA abundance carry substantial uncertainty. Access to well‐characterized human samples, both from healthy and cancer populations, remains limited and will be required to address this question. These limitations represent avenues for refinement of the proposed QSP strategy as additional data become available. Finally, whether these techniques offer an advantage over the commonly practised use of disease phenotype (e.g., tumour size reduction) needs to be assessed if this approach is to be used in a clinical setting. Such validation may require patient stratification and monitoring of the impact of dose adjustment against long‐term clinical outcomes.

## AUTHOR CONTRIBUTIONS

Zubida M. Al‐Majdoub, Armin Sepp, Amin Rostami‐Hodjegan and Brahim Achour wrote the manuscript; Brahim Achour designed the research; Amin Rostami‐Hodjegan and Brahim Achour acquired the funding; Zubida M. Al‐Majdoub, Armin Sepp and Brahim Achour performed the research; Armin Sepp and Brahim Achour analysed the data.

## CONFLICT OF INTEREST STATEMENT

Certara Ltd. holds several patents related to Liquid Biopsy, and Amin Rostami‐Hodjegan and Armin Sepp (via employment) and Brahim Achour (via consultancy) are linked to Certara's activities in this space. All other authors declared no competing interests for this work.

## Supporting information


**Table S1.** Demographic and clinical information for the matched liver and plasma donors.
**Table S2.** Demographic information related to healthy plasma donors.
**Table S3.** Expression of PD targets related to cancer and organ health in liquid biopsy.
**Table S4.** Protein abundance of PD targets related to cancer and organ health in membrane fractions from histologically normal liver tissue collected from metastatic liver cancer patients.
**Figure S1.** QSP model structure to simulate the PK and PD interactions between EGF (ligand), EFGR (EGF receptor) and cetuximab (mAb targeting EGFR) encompassing interstitial, endosomal and vascular compartments.
**Figure S2.** Distribution of EGF and EGFR expression data in plasma extracellular vesicles from healthy and cancer cohorts.
**Figure S3.** Cross‐correlation between EGF and EGFR expression.
**Figure S4.** Tumour EGFR receptor occupancy (RO) with its natural ligand EGF over tested ranges of relative expression measured in the present study in the absence of the mAb.
**Figure S5.** Tumour EGFR occupancy with its natural ligand EGF over tested ranges of relative expression measured in the present study in the presence of the mAb with different affinities of binding and at different doses of the mAb.
**Figure S6.** Tumour EGFR occupancy by the mAb at different affinities and at different doses.
**Figure S7.** Tumour EGFR occupancy by the mAb and EGF at different affinities and at different doses of the mAb.
**Figure S8.** Cross‐correlation between measured protein abundance values in liver membrane QC samples.

## Data Availability

Data are available on request from the corresponding author.
