# Supplementary material for: Monitoring pharmacodynamic and molecular drug targets in liquid biopsy: Exploratory study in liver cancer with modelling of EGFR Receptor engagement
Source: Br J Clin Pharmacol. 2026 Jul 14;92(11):3902–13. doi: 10.1002/bcp.70674 (PMC13714612; doi:10.1002/bcp.70674)
Supplement: Supplementary file 1 [file BCP-92-3902-s001.docx]

Supporting Information

**Monitoring Pharmacodynamic and Molecular Drug Targets in Liquid Biopsy: Exploratory Study in Liver Cancer with Modelling of EGFR Receptor Engagement**

Zubida M. Al-Majdoub, Armin Sepp, Amin Rostami-Hodjegan, Brahim Achour*

*Correspondence to: Dr B Achour; email: [brahim.achour@manchester.ac.uk](mailto:brahim.achour@manchester.ac.uk)

**Supporting Information Content**

**Supplementary Methods**

**Table S1.** Demographic and clinical information for the matched liver and plasma donors.

**Table S2.** Demographic information related to healthy plasma donors.

**Table S3.** Expression of PD targets related to cancer and organ health in liquid biopsy.

**Table S4.** Protein abundance of PD targets related to cancer and organ health in membrane fractions from histologically normal liver tissue collected from metastatic liver cancer patients.

**Figure S1.** QSP model structure to simulate the PK and PD interactions between EGF (ligand), EFGR (EGF receptor) and cetuximab (mAb targeting EGFR) encompassing interstitial, endosomal and vascular compartments.

**Figure S2.** Distribution of EGF and EGFR expression data in plasma extracellular vesicles from healthy and cancer cohorts.

**Figure S3.** Cross-correlation between EGF and EGFR expression.

**Figure S4.** Tumour EGFR receptor occupancy (RO) with its natural ligand EGF over tested ranges of relative expression measured in the present study in the absence of the mAb.

**Figure S5.** Tumour EGFR occupancy with its natural ligand EGF over tested ranges of relative expression measured in the present study in the presence of the mAb with different affinities of binding and at different doses of the mAb.

**Figure S6.** Tumour EGFR occupancy by the mAb at different affinities and at different doses.

**Figure S7.** Tumour EGFR occupancy by the mAb and EGF at different affinities and at different doses of the mAb.

**Figure S8.** Cross-correlation between measured protein abundance values in liver membrane QC samples.

**Supplementary Methods**

***Model parameterization***

A QSP model of EGF and EGFR interaction in the absence and presence of cetuximab was built using the SimBiology toolbox in MATLAB 2023b. An outline of the interactions taking place in the compartments of the model is shown in **Fig S1**. The model was based on the standard full PBPK tissue layout scheme introduced for mAbs by Shah and Betts^1^ and was modified to accommodate two-pore circulation of proteins of any size, as reported previously by Sepp et al.,^2^ with several modifications, as detailed in Supplementary Methods. The central plasma pool is split into venous and arterial compartments, while small fractions of skin and skeletal muscle are separated as subcutaneous and intramuscular dosing compartments with dedicated draining lymph nodes, according to Stader et al.^3^ Solid tumour penetration follows the diffusion-driven well-stirred formalism developed by Thurber et al.^4^ Organ volumes, plasma flow rates and two-pore parameters of the model were based on the BioDMET database values reported by Graf et al.,^5^ while pore sizes are from Rippe and Haraldsson.^6^

Population average EGFR tissue concentrations were based on quantitative protein mass spectrometric values (in parts per million), as reported in the PaxDB database.^7^ These were converted into molar values, as described by Sepp and Muliaditan (values listed in Table 2 of the cited reference).^8^ A value of 1 million EGFR receptors per tumour cell^9^ were used in the solid tumour compartment, which contains 100 million cells per mL volume.^10^ The plasma concentration of soluble EGFR was set at 1.5 nM, as calculated from the plasma proteomics data.^8^ The membrane EGFR extracellular domain shedding rate constant of 1.12×10^-4^ h^-1^ was empirically adjusted to result in 1.5 nM steady-state conditions of soluble EGFR in the healthy population. Highly variable levels of normal healthy human EGF plasma concentration have been reported, varying from 2 pM to 51 pM,^11^ and even up to 750 pM.^12,13^ The median value of 51 pM was adopted for modelling. EGF secretion was postulated to occur in the central plasma compartment at a constant rate that was empirically adjusted to 0.516 nmol/h to achieve a 51 pM steady-state value in the healthy population.

Average healthy and patient EV-derived cfRNA counts for EGF and EGFR measured in the present study were assumed to be proportional to the average plasma concentration values for EGF and EGFR protein throughout their ranges of variation. The measured values were used to estimate variability in each cohort and changes in disease from healthy baseline. EGF and EGFR mRNA levels in plasma EVs from the healthy and patient subgroups were fitted to lognormal distribution to calculate geometric means and confidence intervals (**Fig S2**). Higher levels of EGF and EGFR RNA were detected in the plasma samples from cancer patients, whereas weak correlation was uncovered between individual EGF and EGFR RNA expression data (**Fig S3**); therefore, EGFR and EGF were not correlated in the model. EGFR was also measured in matching liver tissue fractions by proteomics to assess correlation between EGFR in plasma EVs and EGFR in liver tissue.

***Model testing and cetuximab simulations***

The QSP model was used to estimate EGFR engagement with EGF or cetuximab under different conditions of EGF and EGFR expression levels in the presence of the mAb at different binding affinities and concentrations. Five EGF expression levels were used to achieve 0.5x, 1x, 3x, 9x and 27x steady-state concentrations of plasma EGF relative to healthy average. Steady-state concentrations of EGF included the variation found in 95% of the patient population. The shedding, internalization and baseline expression rate constants of membrane EGFR were taken from the PBPK model previously established for cetuximab.^8^ Only the membrane EGFR expression rate constant was proportionally adjusted to achieve steady-state soluble EGFR concentration values that varied 0.5x, 1x, 2.5x, 6.25x or 15.6x from healthy average at steady state. The concentrations of tissue and plasma EGFR included the variation found in 95% of the patient population. Steady-state tissue and plasma concentrations of EGF and soluble EGFR at 100 days were used as the starting point for subsequent dose and target engagement modelling. In total, 500 simulations were conducted:

- Five EGF expression levels ranging from 0.5x to 27x relative to healthy mean (0.5x, 1x, 3x, 9x, 27x) to cover 95% of the observed range
- Five EGFR expression levels ranging from 0.5x to 15.6x relative to healthy mean (0.5x, 1x, 2.5x, 6.25x, 15.6x) to cover 95% of the observed range
- Four levels of anti-EGFR antibody affinity for EGFR (Kd = 1.9 pM, 19 pM, 190 pM, 1.9 nM). mAb affinity for soluble EGFR was assumed to be equal to that for membrane-bound EGFR. Antibody-EGFR internalization constant of 0.0049 h^-1^ was adopted from our previous PBPK model.^8^ The same value was also adopted for the EGF-EGFR complex and for free EGFR. The total cell surface concentration of EGFR was modelled as constant in time
- Five dosing regimens for five repeat Q4W IV infusion scenarios averaging 0, 0.5, 1, 3, 10 mg/kg were simulated

Highly variable values for EGF binding to EGFR are reported in the literature,^14,15^ from picomolar high affinity to nanomolar low affinity. The model used Kd = 200 pM for EGF binding to membrane EGFR and Kd = 8 nM for EGF binding to soluble EGFR, to reflect the likely loss of avidity effect in soluble complexes.

The developed model was then used to test a standard dosing regimen based on the package insert of cetuximab (ERBITUX) as indicated for colorectal cancer patients (400 mg/m^2^ followed by 250 mg/m^2^ weekly until steady state is reached), while varying the concentration of EGFR and keeping the anti-EGFR antibody affinity for EGFR at Kd = 1.9 nM.

**Table S1** Demographic and clinical information for the matched liver and plasma donors.

| **ID** | **Donor** | **Sex** | **Age**  (years) | **Ethnicity** | **Height** (m) | **Weight** (kg) | **BMI** (kg/m^2^) | **Diagnosis** | **Medical history** | **Drinking** | **Smoking** |
| --- | --- | --- | --- | --- | --- | --- | --- | --- | --- | --- | --- |
| **1** | **662** | F | 76 | Caucasian | - | 66 | - | Gallbladder cancer | Gallbladder cancer, high BP | - | - |
| **2** | **697** | M | 66 | Caucasian | 1.83 | 114 | 34 | Liver metastasis (bowel sigmoid origin) | Bowel tumour, psoriasis, hernia, high BP | Yes | Yes |
| **3** | **728** | M | 69 | Caucasian | 1.72 | 77 | 26 | Liver metastasis (colorectal origin) | Anterior resection for rectal tumour, tonsillectomy, appendectomy, epilepsy | - | No |
| **4** | **746** | M | 85 | Caucasian | 1.78 | 75 | 24 | Liver metastasis (colorectal origin) | Laparoscopic hemicolectomy, SCC scalp, hypothyroidism, hypertension, COPD | Moderate | Ex-smoker |
| **5** | **766** | F | 72 | - | - | - | - | Liver metastasis (colorectal origin) | Arthritis, high BP, diabetes, hiatus hernia, heart burn, reflux | - | - |
| **6** | **794** | F | 71 | Caucasian | 1.69 | 64 | 22 | Liver metastasis (colorectal origin) | Hemicolectomy, high BP, depression | No | No |
| **7** | **806** | M | 56 | Caucasian | 1.80 | 64 | 20 | Liver metastasis (colorectal origin) | Anterior resection, adenocarcinoma, pancreatitis | - | Yes |
| **8** | **813** | F | 60 | Caucasian | 1.66 | 115 | 42 | Liver metastasis (colorectal origin) | Anterior resection, hemicolectomy, COPD, diabetes | - | Ex-smoker |
| **9** | **818** | M | 58 | Caucasian | 1.78 | 69 | 22 | Liver metastasis (colorectal origin) | Sigmoid adenocarcinoma | Yes | Ex-smoker |
| **10** | **829** | M | 78 | Caucasian | 1.75 | 74 | 24 | Liver metastasis (colorectal origin) | Rectal cancer | - | No |
| **11** | **855** | F | 79 | Caucasian | 1.57 | 48 | 19 | Hepatocellular carcinoma | Angina, diabetes | Rare | Ex-smoker |
| **12** | **1071** | M | 71 | Caucasian | - | - | - | Liver metastasis (colorectal origin) | Hemicolectomy for cancer | - | No |
| **13** | **1304** | M | 57 | Caucasian | 1.74 | 70 | 23 | Liver metastasis (colorectal origin) | Arthritis, hemicolectomy, joint pain in knee, high BP, asthma | Yes | No |
| **14** | **1372** | F | 65 | Caucasian | 1.50 | 47 | 21 | Liver metastasis (colorectal origin) | Anterior resection, asthma, hypothyroidism, breast cancer, lumpectomy, cholecystectomy | - | Ex-smoker, e-cigarette user |
| **15** | **493** | F | 50 | Caucasian | - | 80 | - | Liver metastasis (colorectal origin) | Previous liver resection, high BP, asthma, reflux, breast cancer, colorectal cancer | - | - |
| **16** | **590** | M | 72 | Caucasian | 1.75 | 98 | 32 | Liver metastasis (colorectal origin) | Asthma, polypectomy, tonsillectomy, hemicolectomy | Yes | Yes, pipe user |
| **17** | **645** | F | 61 | - | 1.53 | 84 | 36 | Liver metastasis (colorectal origin) | Asthma, upper rectal adenocarcinoma, anterior resection | No | No |
| **18** | **646** | M | 44 | - | 1.72 | 90 | 30 | Liver metastasis (colorectal origin) | High BP, primary sigmoid adenocarcinoma | - | No |
| **19** | **674** | F | 68 | Caucasian | 1.50 | 60 | 27 | Liver metastasis (colorectal origin) | Recto-sigmoid cancer | - | No |
| **20** | **682** | M | 60 | Caucasian | 1.90 | 118 | 33 | Liver metastasis (colorectal origin) | MI, gout, high BP, primary rectal cancer | - | Ex-smoker |
| **21** | **756** | M | 57 | Caucasian | 1.62 | 71 | 27 | Liver metastasis (bowel cancer) | IHD, COPD, hypothyroidism, bowel cancer, anterior resection, previous liver resection | Moderate | Ex-smoker |
| **22** | **781** | M | 57 | - | - | 100 | 31 | Liver metastasis (colorectal origin) | Anterior resection, kidney stones | - | No |
| **23** | **734** | F | 64 | Caucasian | 1.68 | 67 | 24 | Liver metastasis (colorectal origin) | Anterior resection, heart murmur | Occasional | No |
| **24** | **755** | M | 63 | Caucasian | 1.74 | 85 | 28 | Liver metastasis (colorectal origin) | Low anterior resection | - | No |
| **25** | **770** | F | 61 | Caucasian | 1.57 | 51 | 21 | Liver metastasis (colorectal origin) | Colorectal tumour resection, high BP | - | - |
| **26** | **389** | F | 52 | Caucasian | 1.62 | 81 | 31 | Liver metastasis (rectal and sigmoid origin) | DVT, asthma, duodenal ulcer, thyroid problem, liver lesions | Occasional | No |
| **27** | **589** | F | 69 | Caucasian | 1.58 | 52 | 21 | Liver metastasis (colorectal adenocarcinoma) | Colon adenocarcinoma, cervical cancer, anxiety, depression | - | Yes |
| **28** | **1063** | M | 77 | Caucasian | 1.70 | 77 | 27 | Hepatocellular carcinoma | Prostate cancer | Yes | Ex-smoker |
| **29** | **1359** | M | 68 | Caucasian | 1.66 | 92 | 33 | Primary liver cancer (cholangiocarcinoma) | Right elbow surgery, hiatus hernia, reflux | Yes | No |

Age at the time of surgery; BP, blood pressure; BMI, body mass index; COPD, chronic obstructive pulmonary disease; DVT, deep vein thrombosis; F, female; IHD, ischemic heart disease; M, male; MI, myocardial infarction; SCC, squamous cell carcinoma; - symbols represent missing information from patient records.

**Table S2.** Demographic information related to healthy plasma donors.

| **ID** | **Donor ID** | **Age**  (years) | **Sex** | **Ethnicity** | **Medical history** |
| --- | --- | --- | --- | --- | --- |
| **101** | **197** | 51 | M | Caucasian | Healthy |
| **102** | **188** | 23 | F | Hispanic | Healthy |
| **103** | **189** | 39 | F | Hispanic | Healthy |
| **104** | **193** | 53 | M | Black | Healthy |
| **105** | **194** | 57 | M | Caucasian | Healthy |
| **106** | **185** | 41 | M | Hispanic | Healthy |
| **107** | **192** | 53 | M | Caucasian | Healthy |

Age (years) at the time of blood collection; F, female; M, male. Plasma was sampled in April 2018.

**Table S3.** Expression of PD targets related to cancer and organ health in liquid biopsy. Expression levels above the limit of detection were normalized to the total number of reads in each sample.

| **Gene name** | **Mean** *^a^* | **SD** *^a^* | ***n*** | **FC** *^b^* |  | **Gene name** | **Mean** *^a^* | **SD** *^a^* | ***n*** | **FC** *^b^* |
| --- | --- | --- | --- | --- | --- | --- | --- | --- | --- | --- |
| AATK | 4.59 | 3.24 | 29 | 1.66 |  | EPHA10 | 0.06 | 0.05 | 3 | 0.65 |
| ABL1 | 15.41 | 6.39 | 29 | 0.73 |  | EPHA2 | 2.33 | 1.27 | 29 | 0.76 |
| ABL2 | 8.21 | 4.22 | 29 | 1.53 |  | EPHA3 | 0.33 | 0.24 | 14 | 0.98 |
| ACACA | 1.54 | 1.18 | 29 | 0.46 |  | EPHA4 | 0.63 | 0.33 | 27 | 0.82 |
| ACACB | 11.30 | 6.39 | 29 | 0.87 |  | EPHA5 | 0.02 | 0.01 | 6 | 1.22 |
| AGTR1 | 0.12 | 0.05 | 6 | - |  | EPHA7 | 0.74 | 0.81 | 17 | 0.33 |
| AGTRAP | 11.07 | 5.12 | 29 | 0.93 |  | EPHB1 | 0.43 | 0.31 | 28 | 0.58 |
| AKT1 | 14.57 | 6.64 | 29 | 0.77 |  | EPHB2 | 0.37 | 0.31 | 23 | 0.66 |
| AKT2 | 44.78 | 17.52 | 29 | 0.52 |  | EPHB3 | 0.17 | 0.24 | 16 | 0.16 |
| AKT3 | 558.60 | 297.84 | 29 | 0.83 |  | EPHB4 | 27.65 | 13.61 | 29 | 2.01 |
| ALPI | 0.12 | 0.07 | 3 | 0.37 |  | EPHB6 | 2.39 | 1.54 | 29 | 0.55 |
| ALPL | 23.03 | 17.24 | 29 | 1.77 |  | ERBB2 | 9.44 | 5.63 | 29 | 0.31 |
| ANGPTL1 | 0.50 | 0.37 | 28 | 1.82 |  | ERBB3 | 0.40 | 0.42 | 24 | 0.60 |
| ANGPTL2 | 0.73 | 0.74 | 28 | 1.09 |  | ERBB4 | 0.21 | 0.19 | 3 | 0.47 |
| ANGPTL3 | 8.25 | 9.01 | 29 | 13.88 |  | FCGRT | 21.77 | 9.23 | 29 | 1.53 |
| ANGPTL4 | 0.70 | 0.76 | 20 | 1.78 |  | FER | 60.84 | 40.92 | 29 | 2.94 |
| ANGPTL5 | 0.09 | 0.02 | 4 | 0.50 |  | FES | 25.66 | 11.87 | 29 | 1.07 |
| ANGPTL6 | 0.18 | 0.14 | 26 | 0.69 |  | FGF21 | 0.03 | 0.01 | 8 | 0.98 |
| ANGPTL7 | 0.05 | 0.02 | 3 | - |  | FGFR1 | 0.39 | 0.63 | 6 | 0.97 |
| AOC2 | 0.31 | 0.31 | 23 | 0.65 |  | FGFR2 | 0.08 | 0.07 | 13 | 1.35 |
| AOC3 | 3.08 | 1.61 | 29 | 1.90 |  | FGFR3 | 0.03 | 0.01 | 9 | 0.72 |
| APC | 9.81 | 4.64 | 29 | 0.72 |  | FGFR4 | 0.42 | 0.40 | 26 | 1.82 |
| APOA1 | 81.61 | 74.76 | 29 | 19.87 |  | FGR | 108.95 | 66.49 | 29 | 0.53 |
| APOA2 | 199.31 | 179.19 | 29 | 41.19 |  | FLCN | 12.39 | 4.38 | 29 | 0.97 |
| APOB | 7.16 | 6.83 | 29 | 18.78 |  | FLT1 | 3.46 | 2.20 | 29 | 1.26 |
| APOC1 | 56.32 | 46.15 | 29 | 9.01 |  | FLT3 | 3.70 | 3.00 | 29 | 0.58 |
| APOE | 91.96 | 63.73 | 29 | 10.64 |  | FLT4 | 5.54 | 3.10 | 29 | 3.17 |
| APOM | 0.41 | 0.24 | 29 | 0.79 |  | FRK | 0.31 | 0.28 | 24 | 1.20 |
| ARAF | 18.11 | 5.98 | 29 | 0.43 |  | FYN | 108.55 | 44.05 | 29 | 0.67 |
| ARID1A | 66.26 | 31.81 | 29 | 0.67 |  | GLP1R | 0.05 | 0.04 | 4 | 0.24 |
| AURKA | 0.42 | 0.45 | 27 | 0.40 |  | GOT1 | 7.09 | 2.60 | 29 | 0.72 |
| AXIN1 | 9.07 | 3.60 | 29 | 0.64 |  | GOT2 | 13.48 | 4.67 | 29 | 0.64 |
| AXIN2 | 0.92 | 0.59 | 29 | 0.46 |  | GPBAR1 | 22.35 | 17.28 | 29 | 0.57 |
| AXL | 0.16 | 0.11 | 29 | 1.28 |  | GPT | 2.25 | 2.00 | 29 | 10.01 |
| B2M | 9233 | 5868.37 | 29 | 4.52 |  | GPT2 | 1.79 | 1.88 | 29 | 1.38 |
| BAX | 75.54 | 26.04 | 29 | 0.90 |  | GPX1 | 1446.64 | 754.85 | 29 | 1.40 |
| BLK | 14.05 | 10.82 | 29 | 0.86 |  | GPX2 | 3.10 | 3.82 | 28 | 16.51 |
| BMX | 6.33 | 7.01 | 29 | 2.76 |  | GPX3 | 19.37 | 14.41 | 29 | 2.05 |
| BRAF | 130.85 | 56.35 | 29 | 0.80 |  | GPX4 | 89.69 | 32.39 | 29 | 0.66 |
| BRCA1 | 2.09 | 1.09 | 29 | 0.76 |  | GPX7 | 3.47 | 1.71 | 29 | 0.97 |
| BRCA2 | 0.86 | 0.64 | 28 | 0.54 |  | GPX8 | 1.17 | 0.64 | 29 | 2.20 |
| BTK | 31.04 | 13.61 | 29 | 0.71 |  | GSG2 | 104.93 | 90.93 | 29 | 3.91 |
| BUB1 | 13.13 | 6.18 | 29 | 1.41 |  | GSR | 42.75 | 17.56 | 29 | 1.04 |
| BUB1B | 3.45 | 1.93 | 29 | 0.98 |  | HCK | 82.74 | 54.35 | 29 | 0.58 |
| CASP1 | 34.91 | 23.63 | 29 | 0.62 |  | HMGCR | 3.37 | 1.31 | 29 | 0.66 |
| CASP10 | 24.05 | 12.64 | 29 | 0.63 |  | HRAS | 2.93 | 1.12 | 29 | 0.48 |
| CASP2 | 43.98 | 16.44 | 29 | 0.66 |  | IFITM1 | 31.7 | 11.76 | 29 | 0.61 |
| CASP3 | 20.10 | 9.20 | 29 | 0.75 |  | IFITM10 | 3.10 | 2.24 | 28 | 2.76 |
| CASP4 | 190.88 | 92.01 | 29 | 0.75 |  | IFITM2 | 187.90 | 156.49 | 29 | 0.55 |
| CASP5 | 0.02 | 0.01 | 9 | 0.45 |  | IFITM3 | 140.83 | 41.91 | 29 | 0.89 |
| CASP6 | 6.45 | 2.77 | 29 | 0.61 |  | IFITM4P | 0.72 | 0.67 | 29 | 4.05 |
| CASP7 | 1.36 | 1.08 | 28 | 0.53 |  | IGF1R | 9.98 | 5.45 | 29 | 0.99 |
| CASP8 | 1.42 | 0.88 | 29 | 0.59 |  | IGF2R | 7.87 | 4.41 | 29 | 0.95 |
| CASP9 | 11.36 | 5.75 | 29 | 0.72 |  | IL10 | 0.79 | 1.04 | 26 | 0.89 |
| CAV1 | 0.30 | 0.23 | 24 | 0.65 |  | IL10RA | 12.81 | 7.33 | 29 | 0.35 |
| CAV2 | 42.26 | 22.41 | 29 | 1.30 |  | IL10RB | 13.25 | 7.89 | 29 | 0.96 |
| CCND1 | 165.41 | 65.51 | 29 | 0.80 |  | IL11 | 0.08 | 0.10 | 9 | 0.65 |
| CCNE1 | 1.09 | 0.60 | 29 | 0.46 |  | IL11RA | 0.81 | 0.64 | 29 | 0.45 |
| CDK4 | 26.55 | 10.14 | 29 | 0.57 |  | IL12A | 0.28 | 0.28 | 25 | 0.40 |
| CDK6 | 18.61 | 8.25 | 29 | 0.52 |  | IL12RB1 | 0.61 | 0.45 | 29 | 0.88 |
| CDKN2A | 0.10 | 0.09 | 25 | 0.75 |  | IL12RB2 | 0.92 | 0.79 | 29 | 0.43 |
| CEACAM5 | 0.42 | 0.31 | 10 | - |  | IL13RA1 | 18.54 | 9.37 | 29 | 1.18 |
| CKB | 17.71 | 9.23 | 29 | 1.24 |  | IL13RA2 | 3.87 | 2.65 | 29 | 3.53 |
| CKMT1B | 0.09 | 0.07 | 3 | - |  | IL15 | 2.51 | 1.23 | 29 | 0.68 |
| CKMT2 | 0.22 | 0.21 | 15 | 0.93 |  | IL15RA | 3.87 | 1.65 | 29 | 0.81 |
| CSF1R | 10.49 | 8.75 | 29 | 0.91 |  | IL16 | 1.52 | 1.06 | 29 | 2.13 |
| CSK | 210.84 | 91.21 | 29 | 0.49 |  | IL17A | 0.03 | 0.02 | 4 | 0.33 |
| CTNNB1 | 182.03 | 87.95 | 29 | 0.70 |  | IL17B | 0.18 | 0.10 | 13 | 1.51 |
| CXCR1 | 23.03 | 27.95 | 29 | 1.45 |  | IL17C | 0.03 | 0.03 | 5 | 0.42 |
| CXCR2 | 93.90 | 101.15 | 29 | 1.01 |  | IL17RA | 1.66 | 0.84 | 29 | 0.63 |
| CXCR4 | 27.52 | 25.03 | 29 | 0.71 |  | IL17RB | 0.16 | 0.16 | 10 | 6.56 |
| CXCR5 | 12.42 | 8.65 | 29 | 0.51 |  | IL17RC | 0.11 | 0.08 | 26 | 0.75 |
| CXCR6 | 1.24 | 1.19 | 29 | 1.76 |  | IL17RD | 0.20 | 0.16 | 24 | 1.46 |
| CXCR7 | 0.34 | 0.28 | 24 | 0.60 |  | IL17RE | 0.21 | 0.17 | 28 | 2.28 |
| DDR1 | 0.15 | 0.14 | 21 | 0.50 |  | IL17REL | 0.07 | 0.05 | 5 | - |
| DDR2 | 5.30 | 2.94 | 29 | 1.16 |  | IL18 | 13.48 | 7.18 | 29 | 0.96 |
| DLC1 | 52.20 | 22.81 | 29 | 1.13 |  | IL18R1 | 0.97 | 0.85 | 29 | 1.37 |
| DPP10 | 0.07 | 0.05 | 6 | 1.85 |  | IL18RAP | 7.78 | 9.37 | 29 | 2.10 |
| DPP3 | 10.60 | 6.32 | 29 | 0.62 |  | IL19 | 0.04 | 0.04 | 14 | 0.02 |
| DPP4 | 4.73 | 2.76 | 29 | 0.49 |  | IL1B | 16.51 | 14.51 | 29 | 0.43 |
| DPP7 | 13.70 | 6.13 | 29 | 0.82 |  | IL1F10 | 0.02 | 0.01 | 8 | 0.02 |
| DPP8 | 6.02 | 3.51 | 29 | 0.52 |  | IL1R1 | 21.82 | 16.31 | 29 | 3.73 |
| DPP9 | 5.88 | 3.11 | 29 | 0.60 |  | IL1R2 | 15.22 | 23.26 | 29 | 5.24 |
| EGFR | 1.50 | 0.97 | 27 | 5.30 |  | IL1RAPL1 | 0.07 | 0.05 | 4 | 5.28 |
| EP300 | 73.89 | 33.03 | 29 | 0.81 |  | IL1RAPL2 | 0.07 | 0.05 | 7 | 6.06 |
| EPHA1 | 0.52 | 0.43 | 23 | 0.35 |  | IL1RL1 | 0.15 | 0.18 | 17 | 1.26 |

**Table S3.** Continued.

| **Gene name** | **Mean** *^a^* | **SD** *^a^* | ***n*** | **FC** *^b^* |  | **Gene name** | **Mean** *^a^* | **SD** *^a^* | ***n*** | **FC** *^b^* |
| --- | --- | --- | --- | --- | --- | --- | --- | --- | --- | --- |
| IL1RL2 | 0.18 | 0.11 | 5 | - |  | MAPK11 | 3.68 | 1.76 | 29 | 0.98 |
| IL1RN | 14.26 | 13.89 | 29 | 0.64 |  | MAPK12 | 9.58 | 4.45 | 29 | 1.40 |
| IL2 | 0.16 | 0.12 | 9 | 0.51 |  | MAPK13 | 11.68 | 7.13 | 29 | 1.23 |
| IL20RA | 0.06 | 0.05 | 11 | 0.28 |  | MAPK14 | 47.66 | 33.70 | 29 | 0.73 |
| IL20RB | 0.42 | 0.28 | 25 | 0.52 |  | MAPK15 | 0.03 | 0.02 | 9 | 0.01 |
| IL21 | 0.17 | 0.14 | 7 | 0.65 |  | MAPK3 | 78.62 | 38.18 | 29 | 0.80 |
| IL21R | 2.65 | 1.57 | 29 | 0.43 |  | MAPK4 | 0.18 | 0.15 | 9 | 1.88 |
| IL21R-AS1 | 0.03 | 0.02 | 11 | 0.73 |  | MAPK6 | 13.20 | 6.94 | 29 | 0.90 |
| IL22RA1 | 0.07 | 0.11 | 9 | 0.77 |  | MAPK7 | 8.53 | 4.34 | 29 | 0.62 |
| IL22RA2 | 0.13 | 0.06 | 5 | 0.79 |  | MAPK8 | 0.28 | 0.21 | 27 | 1.14 |
| IL23A | 0.41 | 0.34 | 27 | 0.27 |  | MAPK9 | 36.87 | 15.95 | 29 | 0.66 |
| IL23R | 0.37 | 0.29 | 16 | 0.81 |  | MATK | 44.56 | 16.06 | 29 | 0.53 |
| IL25 | 0.03 | 0.01 | 14 | 0.09 |  | MC4R | 63.52 | 55.42 | 29 | 3.56 |
| IL26 | 0.07 | 0.04 | 5 | 0.48 |  | MCC | 15.06 | 5.91 | 29 | 1.17 |
| IL27 | 1.87 | 1.88 | 27 | 1.58 |  | MERTK | 2.50 | 1.38 | 29 | 1.19 |
| IL27RA | 7.94 | 3.83 | 29 | 0.63 |  | MET | 0.69 | 0.49 | 29 | 0.76 |
| IL28RA | 0.74 | 0.68 | 29 | 0.87 |  | MLH3 | 1.98 | 0.98 | 29 | 0.45 |
| IL2RA | 1.27 | 1.66 | 29 | 0.73 |  | MSH2 | 11.09 | 4.53 | 29 | 0.48 |
| IL2RB | 17.65 | 13.04 | 29 | 0.44 |  | MST1R | 0.20 | 0.09 | 10 | 0.30 |
| IL2RG | 28.92 | 14.02 | 29 | 0.33 |  | MTOR | 15.59 | 7.30 | 29 | 0.53 |
| IL3 | 0.10 | 0.08 | 3 | 0.59 |  | MUC1 | 0.20 | 0.23 | 21 | 0.69 |
| IL31 | 0.02 | 0.01 | 7 | 1.47 |  | MUSK | 0.42 | 0.59 | 5 | - |
| IL31RA | 0.18 | 0.27 | 13 | 0.76 |  | MYC | 25.22 | 13.19 | 29 | 0.46 |
| IL32 | 151.25 | 61.75 | 29 | 0.32 |  | NPC1L1 | 0.24 | 0.23 | 16 | 1.48 |
| IL33 | 28.09 | 19.32 | 29 | 4.00 |  | NPR1 | 2.39 | 1.42 | 29 | 1.80 |
| IL34 | 0.60 | 0.38 | 29 | 0.57 |  | NPR2 | 0.80 | 0.72 | 27 | 1.68 |
| IL36A | 0.40 | 0.75 | 7 | 0.91 |  | NPR3 | 2.90 | 1.39 | 29 | 1.54 |
| IL36B | 0.15 | 0.27 | 5 | 0.56 |  | NR1H4 | 4.71 | 5.45 | 28 | 9.94 |
| IL37 | 0.03 | 0.03 | 5 | 0.04 |  | NR3C2 | 26.55 | 13.31 | 29 | 0.43 |
| IL3RA | 0.84 | 0.46 | 29 | 0.69 |  | NR5A2 | 14.09 | 9.64 | 29 | 1.85 |
| IL4 | 0.13 | 0.13 | 8 | 0.55 |  | NRAS | 44.67 | 17.55 | 29 | 0.48 |
| IL4I1 | 0.17 | 0.10 | 29 | 0.11 |  | NTRK1 | 0.24 | 0.24 | 8 | 0.77 |
| IL4R | 13.70 | 9.06 | 29 | 1.01 |  | NTRK3 | 0.57 | 0.47 | 17 | 1.27 |
| IL5 | 0.03 | 0.03 | 20 | 0.03 |  | P4HB | 52.92 | 17.06 | 29 | 0.95 |
| IL5RA | 0.66 | 0.59 | 26 | 0.96 |  | PAK1 | 81.4 | 46.15 | 29 | 0.83 |
| IL6 | 1.64 | 2.09 | 29 | 1.38 |  | PAK2 | 225.62 | 149.73 | 29 | 1.87 |
| IL6R | 9.69 | 5.52 | 29 | 1.01 |  | PAK3 | 0.10 | 0.07 | 15 | 0.65 |
| IL6ST | 29.91 | 14.84 | 29 | 7.92 |  | PAK4 | 17.19 | 6.34 | 29 | 0.76 |
| IL7 | 2.42 | 1.40 | 29 | 0.44 |  | PAK6 | 0.71 | 0.67 | 29 | 0.54 |
| IL7R | 33.83 | 20.32 | 29 | 0.26 |  | PARP1 | 93.05 | 44.14 | 29 | 0.53 |
| IL8 | 25.14 | 38.99 | 29 | 2.12 |  | PARP2 | 0.79 | 0.61 | 18 | 0.66 |
| IL9 | 0.08 | 0.06 | 12 | 3.42 |  | PDCD1 | 1.48 | 1.45 | 29 | 1.31 |
| IL9R | 0.09 | 0.09 | 6 | 0.51 |  | PDGFRA | 2.11 | 2.27 | 26 | 9.47 |
| ILF2 | 51.65 | 20.9 | 29 | 0.46 |  | PDGFRB | 1.39 | 1.07 | 29 | 0.88 |
| ILF3 | 61.23 | 25.37 | 29 | 0.67 |  | PDGFRL | 0.26 | 0.22 | 18 | 1.40 |
| ILK | 136.68 | 77.20 | 29 | 1.26 |  | PDIA3 | 111.88 | 52.56 | 29 | 1.28 |
| INSR | 5.31 | 4.50 | 29 | 1.37 |  | PDIA4 | 0.11 | 0.07 | 29 | 0.15 |
| INSRR | 0.07 | 0.06 | 7 | 0.72 |  | PDIA5 | 52.98 | 50.75 | 29 | 2.62 |
| IRAK4 | 3.12 | 1.50 | 29 | 0.61 |  | PDIA6 | 55.11 | 25.04 | 29 | 0.84 |
| ITK | 24.13 | 12.04 | 29 | 0.31 |  | PGR | 1.53 | 1.02 | 28 | 1.08 |
| JAK1 | 125.53 | 53.63 | 29 | 0.79 |  | PIK3CA | 16.05 | 8.98 | 29 | 0.82 |
| JAK2 | 31.78 | 14.26 | 29 | 1.03 |  | PKDCC | 0.77 | 0.55 | 29 | 0.98 |
| JAK3 | 24.23 | 11.39 | 29 | 0.63 |  | PLA2G2A | 1.67 | 4.11 | 28 | 2.24 |
| KDR | 19.76 | 10.41 | 29 | 1.32 |  | PLCG1 | 39.46 | 14.49 | 29 | 0.60 |
| KIT | 0.38 | 0.28 | 21 | 0.88 |  | PLK1 | 3.88 | 2.63 | 29 | 0.77 |
| KRAS | 23.81 | 12.62 | 29 | 0.57 |  | PLK2 | 12.35 | 5.09 | 29 | 0.48 |
| KRT19 | 2.12 | 1.60 | 29 | 1.17 |  | PLK3 | 4.00 | 2.05 | 29 | 0.62 |
| KSR1 | 0.32 | 0.31 | 21 | 0.46 |  | PLK4 | 1.01 | 0.67 | 29 | 0.62 |
| KSR2 | 1.33 | 3.84 | 11 | 20.12 |  | PMS1 | 2.51 | 1.36 | 29 | 0.41 |
| LCK | 0.66 | 0.51 | 26 | 0.20 |  | PMS2 | 4.79 | 2.67 | 29 | 0.58 |
| LGALS1 | 150.39 | 74.28 | 29 | 0.58 |  | PNPLA3 | 0.82 | 0.70 | 22 | 0.18 |
| LGALS12 | 9.36 | 5.73 | 29 | 1.35 |  | PNPLA6 | 10.31 | 5.51 | 29 | 0.50 |
| LGALS14 | 0.04 | 0.01 | 4 | 0.39 |  | PPARA | 13.55 | 4.78 | 29 | 0.83 |
| LGALS17A | 0.08 | 0.10 | 9 | 0.34 |  | PPARD | 12.84 | 6.09 | 29 | 0.59 |
| LGALS2 | 5.33 | 4.10 | 29 | 0.25 |  | PPARG | 0.06 | 0.04 | 24 | 0.04 |
| LGALS3 | 65.82 | 38.06 | 29 | 0.46 |  | PRKCA | 45.46 | 25.17 | 29 | 1.02 |
| LGALS4 | 1.81 | 2.35 | 29 | 0.64 |  | PRKCB | 93.39 | 41.67 | 29 | 0.65 |
| LGALS7B | 0.18 | 0.11 | 9 | 0.92 |  | PRKCD | 22.31 | 10.40 | 29 | 0.78 |
| LGALS8 | 26.07 | 11.10 | 29 | 0.91 |  | PRKCE | 6.53 | 4.28 | 29 | 0.93 |
| LGALS9 | 101.15 | 65.13 | 29 | 0.64 |  | PRKCH | 12.86 | 6.50 | 29 | 0.41 |
| LGALS9B | 0.39 | 0.31 | 28 | 0.30 |  | PRKCI | 64.00 | 60.86 | 29 | 3.00 |
| LGALS9C | 3.18 | 2.84 | 29 | 0.57 |  | PRKCQ | 19.58 | 10.65 | 29 | 0.53 |
| LGALSL | 105.89 | 68.92 | 29 | 2.74 |  | PRKCZ | 4.14 | 1.79 | 29 | 0.51 |
| LMTK2 | 5.80 | 2.92 | 29 | 0.86 |  | PTEN | 141.54 | 74.54 | 29 | 0.78 |
| LMTK3 | 0.73 | 0.56 | 25 | 0.30 |  | PTK2 | 204.13 | 107.59 | 29 | 0.77 |
| LOXL1 | 0.03 | 0.02 | 19 | 1.29 |  | PTK2B | 100.24 | 46.54 | 29 | 0.53 |
| LOXL2 | 0.82 | 0.38 | 27 | 0.88 |  | PTK6 | 0.52 | 0.41 | 25 | 0.92 |
| LOXL3 | 1.66 | 1.21 | 29 | 2.06 |  | PTK7 | 2.35 | 2.21 | 29 | 1.77 |
| LOXL4 | 0.14 | 0.14 | 17 | 1.63 |  | PTPN12 | 178.04 | 97.50 | 29 | 1.21 |
| LTK | 1.48 | 0.89 | 28 | 1.18 |  | PTPRJ | 71.10 | 78.13 | 29 | 2.25 |
| LYN | 188.5 | 92.35 | 29 | 0.62 |  | PTRF/CAVIN1 | 285.28 | 119.55 | 29 | 0.97 |
| MAP2K1 | 41.94 | 17.93 | 29 | 0.57 |  | RAD54B | 2.75 | 2.10 | 29 | 0.80 |
| MAP2K2 | 89.48 | 40.72 | 29 | 1.41 |  | RAF1 | 11.20 | 6.87 | 29 | 0.48 |
| MAP3K5 | 21.73 | 9.88 | 29 | 0.91 |  | RET | 0.67 | 0.59 | 28 | 1.11 |
| MAPK1 | 47.15 | 20.52 | 29 | 0.64 |  | ROR1 | 0.62 | 1.27 | 22 | 1.45 |
| MAPK10 | 4.61 | 4.30 | 29 | 5.65 |  | ROR2 | 0.09 | 0.10 | 15 | 0.38 |

**Table S3.** Continued.

| **Gene name** | **Mean** *^a^* | **SD** *^a^* | ***n*** | **FC** *^b^* |
| --- | --- | --- | --- | --- |
| RYK | 16.73 | 7.47 | 29 | 0.55 |
| SCD | 21.95 | 14.44 | 29 | 3.03 |
| SDPR/CAVIN2 | 272.08 | 190.60 | 29 | 5.69 |
| SEPT9 | 155.19 | 78.42 | 29 | 0.52 |
| SERPINH1 | 8.25 | 3.55 | 29 | 1.01 |
| SRC | 23.41 | 14.98 | 29 | 1.40 |
| STAT1 | 105.54 | 52.33 | 29 | 0.51 |
| STAT2 | 38.49 | 19.70 | 29 | 0.60 |
| STAT3 | 128.20 | 58.70 | 29 | 0.97 |
| STAT4 | 4.38 | 2.03 | 29 | 0.34 |
| STAT5A | 57.57 | 23.16 | 29 | 0.85 |
| STAT5B | 63.24 | 29.85 | 29 | 0.51 |
| STAT6 | 112.64 | 47.37 | 29 | 0.63 |
| STRAP | 30.98 | 13.53 | 29 | 0.65 |
| STYK1 | 0.96 | 0.66 | 28 | 0.59 |
| SYK | 111.40 | 44.51 | 29 | 0.67 |
| TEC | 1.97 | 1.45 | 29 | 0.53 |
| TEK | 7.55 | 3.74 | 29 | 1.73 |
| TEX10 | 2.03 | 1.05 | 29 | 0.43 |
| TEX11 | 0.14 | 0.09 | 3 | 2.24 |
| TEX14 | 0.18 | 0.18 | 9 | 0.67 |
| TEX15 | 0.23 | 0.23 | 18 | 1.45 |
| TEX2 | 5.89 | 2.70 | 29 | 1.01 |
| TEX22 | 0.02 | 0.01 | 11 | 0.96 |
| TEX26 | 0.21 | 0.19 | 3 | - |
| TEX261 | 5.40 | 2.93 | 29 | 0.65 |
| TEX264 | 11.73 | 5.10 | 29 | 0.55 |
| TEX28 | 0.16 | 0.08 | 4 | 0.43 |
| TEX29 | 0.75 | 1.74 | 24 | 0.44 |
| TEX30 | 6.16 | 3.54 | 29 | 0.86 |
| TEX33 | 0.014 | 0.005 | 4 | 0.12 |
| TEX35 | 0.10 | 0.15 | 8 | 0.21 |
| TEX9 | 38.38 | 19.13 | 29 | 1.28 |
| TGFBR1 | 7.53 | 4.41 | 29 | 0.43 |
| THRB | 10.67 | 6.80 | 29 | 3.15 |
| TIE1 | 1.70 | 0.94 | 29 | 2.40 |
| TLR1 | 6.10 | 6.37 | 29 | 0.71 |
| TLR10 | 0.56 | 0.37 | 26 | 0.51 |
| TLR2 | 8.39 | 6.53 | 29 | 1.60 |
| TLR3 | 0.41 | 0.32 | 28 | 0.75 |
| TLR4 | 38.6 | 33.39 | 29 | 1.22 |
| TLR5 | 0.28 | 0.28 | 25 | 0.79 |
| TLR6 | 0.80 | 0.94 | 29 | 0.52 |
| TLR7 | 0.76 | 0.83 | 29 | 0.26 |
| TLR8 | 1.46 | 1.12 | 29 | 1.13 |
| TLR9 | 1.78 | 1.34 | 29 | 0.80 |
| TMX3 | 4.60 | 2.46 | 29 | 0.66 |
| TNC | 0.59 | 0.64 | 25 | 2.37 |
| TNK1 | 0.28 | 0.30 | 12 | 0.89 |
| TNK2 | 65.31 | 27.83 | 29 | 0.97 |
| TNNI3K | 0.31 | 0.50 | 8 | 13.19 |
| TP53 | 87.70 | 38.14 | 29 | 0.66 |
| TXK | 26.74 | 14.75 | 29 | 0.40 |
| TYK2 | 20.53 | 8.64 | 29 | 0.70 |
| TYRO3 | 37.50 | 63.35 | 29 | 14.58 |
| VEGFB | 23.07 | 9.05 | 29 | 0.52 |
| VEGFC | 3.69 | 2.33 | 29 | 0.78 |
| YES1 | 54.42 | 24.51 | 29 | 1.19 |
| ZAK | 5.19 | 2.56 | 29 | 0.56 |
| ZAP70 | 4.26 | 2.57 | 29 | 0.29 |

*^a^* mean (and standard deviation, SD) of expression levels (in units of reads per million, RPM) in cancer plasma-derived extracellular vesicles (*n* = 29); *^b^* fold change (FC) from baseline in healthy donors (*n* = 7); - under FC means the target was not detected in healthy samples. Targets detected only in healthy samples (not listed on the table): IL12B, TEX101.

**Table S4.** Protein abundance of PD targets related to cancer and organ health in membrane fractions from histologically normal liver tissue collected from metastatic liver cancer patients (*n* = 29). Abundance is normalized to total membrane protein (pmol/mg membrane protein).

| **Gene/protein** | **Subcellular location** | **PD relevance** | **Abundance (pmol/mg protein)** | | | |
| --- | --- | --- | --- | --- | --- | --- |
|  |  |  | **Mean** | **SD** | **Median** | ***n*** |
| ACACB | Mitochondria | PD marker | 0.48 | 0.54 | 0.22 | 25 |
| ALPL | PM, mitochondria | Organ health, disease marker | 0.22 | 0.18 | 0.14 | 12 |
| AOC3 | PM | Organ health, drug target | 2.16 | 2.61 | 1.52 | 27 |
| APOA1 ^§^* | Secreted | Organ health | 162.47 | 109.15 | 145.53 | 29 |
| APOA2 ^§^* | Secreted | Organ health | 32.93 | 19.27 | 26.87 | 12 |
| APOB ^§^* | Secreted, cytoplasm | Organ health, drug target | 1.16 | 0.97 | 0.76 | 28 |
| APOC1 ^§^* | Secreted | Organ health | 34.79 | 32.02 | 28.63 | 17 |
| APOE | Secreted, EVs, endosome | Organ health | 13.81 | 6.29 | 13.59 | 28 |
| B2M | PM, secreted | Disease marker, immune response | 37.45 | 38.72 | 29.7 | 28 |
| BAX | Mitochondria, cytoplasm, nucleus | Apoptosis marker | 1.19 | 1.40 | 0.66 | 11 |
| CAV1 | Golgi apparatus, PM | Organ health | 4.15 | 1.44 | 4.06 | 18 |
| CAV2 | Cytoplasm, nucleus, Golgi apparatus | Organ health | 6.53 | 2.13 | 6.49 | 22 |
| CTNNB1 | Cell junction, PM, nucleus, cytoplasm | Organ health | 0.52 | 0.37 | 0.53 | 27 |
| DPP4 | Cell junction, PM, secreted | Drug target, immune response | 0.65 | 0.44 | 0.58 | 24 |
| DPP7 (DPP2) | Secreted, lysosome | Disease marker, organ health, immune response | 0.17 | 0.05 | 0.17 | 4 |
| EGFR | PM, ER, Golgi apparatus, nucleus, endosome | RTK, Disease marker, drug target | 0.64 | 0.40 | 0.48 | 26 |
| GOT1* | Cytoplasm | Organ health, disease marker | 4.26 | 6.45 | 2.88 | 28 |
| GOT2 | PM, mitochondria | Organ health, disease marker | 17.54 | 14.72 | 13.19 | 29 |
| GPT | ER | Organ health, disease marker | 3.53 | 2.93 | 2.58 | 27 |
| GPX1 | Mitochondria, cytoplasm | Organ health, disease marker | 4.43 | 8.57 | 1.08 | 6 |
| GSR | Mitochondria, cytoplasm | Disease marker, drug target | 0.32 | 0.23 | 0.21 | 6 |
| IGF2R | Golgi apparatus, endosome | Growth factor receptor, disease marker | 0.35 | 0.22 | 0.35 | 25 |
| ILF2 | Cytoplasm, nucleus | PD marker, immune response | 1.34 | 1.82 | 0.63 | 26 |
| ILF3 | Cytoplasm, nucleus | Disease marker, immune response | 0.55 | 0.35 | 0.65 | 12 |
| ILK | Cell junction, PM, nucleus, cytoplasm | Protein kinase, disease marker | 1.16 | 0.94 | 0.97 | 21 |
| LGALS1* | Cytoplasm, secreted | Apoptosis marker, immune regulation | 40.50 | 41.38 | 24.78 | 27 |
| LGALS4 | Cytosol, secreted, PM | Apoptosis marker | 4.58 | 6.74 | 1.74 | 24 |
| LGALS8* | Cytoplasm | Immune regulation | 0.32 | 0.03 | 0.31 | 3 |
| LGALS9/9C | Cytoplasm, nucleus, secreted | Apoptosis marker, immune regulation | 0.88 | 0.38 | 0.72 | 11 |
| MAP2K1/2 | Cytoplasm, nucleus, membrane | Protein kinases, drug targets | 0.55 | 0.35 | 0.36 | 13 |
| P4HB (PDIA1) | ER, ER lumen, PM | Organ health, disease marker | 308.00 | 105.69 | 274.39 | 29 |
| PDIA3 | ER, ER lumen | PD marker, organ health | 121.97 | 52.49 | 120.55 | 29 |
| PDIA4 | ER lumen | PD marker, organ health | 41.63 | 23.48 | 36.22 | 29 |
| PDIA5 | ER lumen | PD marker, organ health | 3.7 | 2.56 | 3.02 | 29 |
| PDIA6 | ER lumen, PM | PD marker, organ health | 80.26 | 33.56 | 84.68 | 29 |
| PLA2G2A | Secreted, PM, mitochondria | Disease marker, drug target | 6.02 | 8.29 | 1.78 | 3 |
| CAVIN1 | PM, ER, caveola, cytoplasm, mitochondria, nucleus | Organ health | 2.13 | 1.46 | 2.11 | 7 |
| CAVIN2 | Caveola, cytoplasm | Organ health | 0.24 | 0.07 | 0.22 | 9 |
| SEPT9* | Cytoplasm | Disease marker, organ health | 0.19 | 0.08 | 0.18 | 3 |
| SERPINH1 | ER lumen | Disease marker, organ health | 1.35 | 1.19 | 0.94 | 20 |
| STAT1 | Cytoplasm, nucleus | Disease marker, organ health | 2.66 | 2.02 | 2.12 | 4 |
| STAT3 | Cytoplasm, nucleus | Disease marker, organ health | 1.14 | 1.61 | 0.42 | 17 |
| TEX264 | ER, cytoplasm, nucleus | Organ health | 0.67 | 0.24 | 0.74 | 12 |
| TMX3 | ER | Organ health | 0.17 | 0.08 | 0.21 | 7 |

^§^ Liver enriched targets. * Targets not associated with the enriched membrane fraction, and therefore, most likely contaminants. Enrichment data was accessed on the Human Protein Atlas; localization data was accessed on the human UniProtKB database; drug target data were accessed on the Ingenuity Pathway Analysis database. Abbreviations: ER, endoplasmic reticulum; EVs, extracellular vesicles; PD, pharmacodynamic; PM, plasma membrane; RTK, receptor tyrosine kinase; SD, standard deviation.

**Figure S1.** QSP model structure to simulate the PK and PD interactions between EGF (ligand), EFGR (EGF receptor) and cetuximab (mAb targeting EGFR) encompassing interstitial, endosomal and vascular compartments. The model includes FcRn recycling, membrane and soluble EGFR in every tissue,^16^ mAb binding membrane and soluble EGFR, EGF,^17^ and possibility of a theoretical mAb binding to EGF. All membrane complexes are subject to target-mediated drug disposition (TMDD), for both EGF and mAb. Each organ includes endosomal and vascular compartments. In vascular and interstitial compartments, EGF is represented as a dark green oval (L), membrane EGFR as a light grey hexagon (mTarget), soluble EGFR is shown as a dark grey oval (sTarget), anti-EGFR mAb is shown as a red quadrangle (Drug), anti-EGF-mAb as a green quadrangle (Drug2). In addition to various complexes: soluble EGFR with EGF (green oval, sTarget_L), soluble EGFR with mAb (dark red oval, sTarget_mAb), membrane EGFR with mAb (purple hexagon, mTarget_mAb), EGF with anti-EGF mAb (light green oval, L_Drug2). Triangles denote protein expression reactions: dark green for EGF (L) and yellow for membrane EGFR. Black circles denote degradation reactions. Yellow round circles denote the binding reactions, and two-pore exchange reactions (when named as v2Pi_x). All soluble proteins are subject to endosomal uptake by macropinocytosis from vascular and interstitial compartments (reactions v2e_x and i2e_x). In the endosomal compartment, yellow grey hexagons denote free FcRn. FcRn can form complexes with free EGFR-binding mAb (red circle, FcRn-Drug) and its complex with soluble EGFR (dark red circle, sTarget_mAb_FcRn), EGF-binding mAb (dark red rectangle, Drug2_FcRn) and its complex with EGF (brown oval, L_Drug2_FcRn). All molecules that are not bound to FcRn, are subject to endosomal degradation, while the FcRn complexes are subject to recycling back to the vascular and interstitial spaces (reactions eiRec_x and evRwx_x).

**(C)**

| **Concentration (RPM)** | **Healthy** | | **Cancer** | |
| --- | --- | --- | --- | --- |
|  | *Mean* ± SD | *Mean _Geo_ (95%CI)* | *Mean* ± SD | *Mean _Geo_ (95%CI)* |
| **EGF** | 2.56 ± 1.30 | 2.24 (1.75;2.87) | 13.07 ± 13.92 | 7.66 (4.97;11.81) |
| **EGFR** | 0.28 ± 0.19 | 0.21 (0.12;0.34) | 1.50 ± 0.97 | 1.23 (0.96;1.59) |

**Figure S2.** Distribution of EGF (**A**) and EGFR (**B**) expression data in plasma extracellular vesicles from healthy and cancer cohorts. Expression data of both ligand and receptor closely follow log-normal distribution. Table (**C**) summarizes the distribution of the expression data. Data presented as means, standard deviations (SD), geometric means and confidence intervals. Abbreviations: RPM, reads per million, CI, confidence interval.

**Figure S3.** Cross-correlation between EGF and EGFR expression (in units of reads per million, RPM) in healthy and cancer cohorts (Pearson’s correlation coefficient, r = 0.27, *p* = 0.08).

**Figure S4.** Tumour EGFR receptor occupancy (RO) with its natural ligand EGF over tested ranges of relative expression measured in the present study in the absence of the mAb. A total of 500 simulations were performed over EGF (0.5x to 27x base level) and EGFR (0.5x to 15.6x base level) expression ranges, which cover 95% of the data. 1x denotes median values for healthy controls. At normal EGF and EGFR levels (1x vs 1x), EGFR occupancy by EGF would be 0.055.

**Figure S5.** Tumour EGFR occupancy with its natural ligand EGF over tested ranges of relative expression measured in the present study in the presence of the mAb with different affinities (decreasing from top to bottom) of binding and at different doses of the mAb (increasing from left to right). 500 simulations were performed over EGF (0.5x to 27x base level) and EGFR (0.5x to 15.6x base level) expression levels that cover 95% of the data. 1x denotes median values for healthy. Affinity for EGFR Kd used four variants (1.9 pM, 19 pM, 190 pM, 1.9 nM). Dosing regimens for continuous infusion scenarios averaging 0, 0.5, 1, 3 10 mg/kg/month. Units: Kd (Molar, M) and dose (mg/kg/month). The mAb competes with EGF and the dose of the mAb is more impactful than affinity of the mAb. There is an EGFR expression range (5- to 10-fold over normal), where EGF is more difficult to replace. This may be related to different rates of tumour penetration by mAbs and EGF- the latter, being smaller, would extravasate much more readily than the bulky mAb. The simulated data suggest that higher doses (1 to 10 mg/kg/month) are likely to be efficacious, which also reflects the clinically relevant range. It is unlikely that affinity maturation will significantly improve efficacy.

**Figure S6.** Tumour EGFR occupancy with the mAb at different affinities and at different doses. The same expression, dose and affinity ranges as **Fig S5** apply. The mAb binds EGFR in a dose- and affinity-dependent manner. Higher dose and higher affinity facilitate receptor binding.

**Figure S7.** Tumour EGFR occupancy with the mAb and EGF at different affinities and at different doses of the mAb. The same expression, dose and affinity ranges as **Fig S5** apply.

**Figure S8.** Cross-correlation between measured protein abundance values in liver membrane QC samples (*n* = 1219 proteins). Correlation was tested for agreement between values (Pearson’s correlation test, r) and for linearity and scatter (linear regression). The dashed line is the line of identity. QC, quality control.

**References**

1.	Shah DK, Betts AM. Towards a platform PBPK model to characterize the plasma and tissue disposition of monoclonal antibodies in preclinical species and human. *J Pharmacokinet Pharmacodyn*. 2012;39(1):67-86. doi:10.1007/s10928-011-9232-2

2.	Sepp A, Meno-Tetang G, Weber A, Sanderson A, Schon O, Berges A. Computer-assembled cross-species/cross-modalities two-pore physiologically based pharmacokinetic model for biologics in mice and rats. *J Pharmacokinet Pharmacodyn*. 2019;46(4):339-359. doi:10.1007/s10928-019-09640-9

3.	Stader F, Liu C, Derbalah A, et al. A Physiologically Based Pharmacokinetic Model Relates the Subcutaneous Bioavailability of Monoclonal Antibodies to the Saturation of FcRn-Mediated Recycling in Injection-Site-Draining Lymph Nodes. *Antibodies*. 2024;13(3):70. doi:10.3390/antib13030070

4.	Thurber GM, Schmidt MM, Wittrup KD. Antibody tumor penetration: transport opposed by systemic and antigen-mediated clearance. *Adv Drug Deliv Rev*. 2008;60(12):1421-1434. doi:10.1016/j.addr.2008.04.012

5.	Graf JF, Scholz BJ, Zavodszky MI. BioDMET: a physiologically based pharmacokinetic simulation tool for assessing proposed solutions to complex biological problems. *J Pharmacokinet Pharmacodyn*. 2012;39(1):37-54. doi:10.1007/s10928-011-9229-x

6.	Rippe B, Haraldsson B. Transport of macromolecules across microvascular walls: the two-pore theory. *Physiol Rev*. 1994;74(1):163-219. doi:10.1152/physrev.1994.74.1.163

7.	Huang Q, Szklarczyk D, Wang M, Simonovic M, von Mering C. PaxDb 5.0: Curated Protein Quantification Data Suggests Adaptive Proteome Changes in Yeasts. *Mol Cell Proteomics*. 2023;22(10):100640. doi:10.1016/j.mcpro.2023.100640

8.	Sepp A, Muliaditan M. Application of quantitative protein mass spectrometric data in the early predictive analysis of membrane-bound target engagement by monoclonal antibodies. *MAbs*. 2024;16(1):2324485. doi:10.1080/19420862.2024.2324485

9.	Gullick WJ, Marsden JJ, Whittle N, Ward B, Bobrow L, Waterfield MD. Expression of epidermal growth factor receptors on human cervical, ovarian, and vulval carcinomas. *Cancer Res*. 1986;46(1):285-292.

10.	Countercurrents Series, Narod SA. Disappearing breast cancers. *Curr Oncol*. 2012;19(2):59-60. doi:10.3747/co.19.1037

11.	Mattila AL, Viinikka L, Saario I, Perheentupa J. Human epidermal growth factor: renal production and absence from plasma. *Regul Pept*. 1988;23(1):89-93. doi:10.1016/0167-0115(88)90424-7

12.	Brzeziński J, Lewiński A. Increased plasma concentration of epidermal growth factor in female patients with non-toxic nodular goitre. *Eur J Endocrinol*. 1998;138(4):388-393. doi:10.1530/eje.0.1380388

13.	Frindik JP, Kemp SF, Elders MJ. ROLE OF GROWTH HORMONE ON PLASMA EPIDERMAL GROWTH FACTOR CONCENTRATIONS. *Pediatr Res*. 1987;21(4):247A-247A. doi:10.1203/00006450-198704010-00480

14.	Krall JA, Beyer EM, MacBeath G. High- and low-affinity epidermal growth factor receptor-ligand interactions activate distinct signaling pathways. *PLoS One*. 2011;6(1):e15945. doi:10.1371/journal.pone.0015945

15.	Ringerike T, Stang E, Johannessen LE, Sandnes D, Levy FO, Madshus IH. High-affinity binding of epidermal growth factor (EGF) to EGF receptor is disrupted by overexpression of mutant dynamin (K44A). *J Biol Chem*. 1998;273(27):16639-16642. doi:10.1074/jbc.273.27.16639

16.	Muliaditan M, Sepp A. Application of quantitative protein mass spectrometric data in the early predictive analysis of target engagement by monoclonal antibodies. *Clin Transl Sci*. 2022;15(7):1634-1643. doi:10.1111/cts.13278

17.	Björkelund H, Gedda L, Andersson K. Comparing the epidermal growth factor interaction with four different cell lines: intriguing effects imply strong dependency of cellular context. *PLoS One*. 2011;6(1):e16536. doi:10.1371/journal.pone.0016536
